# From income inequality to social inequity: impact on health levels in an international efficiency comparison panel

**DOI:** 10.1186/s12889-021-10395-7

**Published:** 2021-04-08

**Authors:** Simone Schenkman, Aylene Bousquat

**Affiliations:** Department of Policies, Management and Health, Faculdade de Saúde Pública - FSP (School of Public Health), Universidade de São Paulo - USP (Sao Paulo University), Av. Dr. Arnaldo 715, Cerqueira César, São Paulo, SP 01246-904 Brazil

**Keywords:** Health equity, Efficiency, Effectiveness, Health systems, Capitalism, Income inequality, Life expectancy at birth, Infant mortality, Data envelopment analysis (DEA), Panel analysis

## Abstract

**Background:**

Health equity, although addressed in several publications dealing with health efficiency analysis, is not easily translated into the operationalization of variables, mainly due to technical difficulties. Some studies provide evidence that it does not influence health outcomes; others demonstrate that its effect is an indirect one, with the hegemony of material living conditions over its social connotation. The aim of this article is to evaluate the role of health equity in determining health outcomes, in an international comparative analysis of the effectiveness and efficiency of health systems.

**Method:**

Fixed Effects Model Panel and Data Envelopment Analysis, a dynamic and network model, in addition to comparative analysis between methods and health impacts. The effect variables considered in the study were life expectancy at birth and infant mortality, in 2010 and 2015, according to the sociocultural regions of the selected countries. Inequity was assessed both economically and socially. The following dimensions were considered: physical and financial resources, health production (access, coverage and prevention) and intersectoral variables: demographic, socioeconomic, governance and health risks.

**Results:**

Both methods demonstrated that countries with higher inequity levels (regarding income, education and health dimensions), associated or not with poverty, are the least efficient, not reaching the potential for effective health outcomes. The outcome *life expectancy at birth* exhibited, in the final model, the following variables: social inequity and per capita health expenditure. The outcome *infant mortality* comprehended the level of education variable, in association with the following healthcare variabels: care seeking due to diarrhea in children under five, births attended by skilled health professionals and the reduction in the incidence of HIV.

**Conclusion:**

The dissociation between the distribution of health outcomes and the overall level of health of the population characterizes a devastating political choice for society, as it is associated with high levels of segregation, disrespect and violence from within. Countries should prioritize health equity, adding value to its resources, since health inequties affect society altogether, generating mistrust and reduced social cohesion.

**Supplementary Information:**

The online version contains supplementary material available at 10.1186/s12889-021-10395-7.

## Background

Health equity, although addressed in several articles and publications about health efficiency analysis, does not usually remain as a relevant result in the empirical studies that propose to test it in their econometric models. In fact, its importance is not translated into the operationalization of variables, which usually primarily reflect the material living conditions, reduced to per capita health expenditure or per capita GDP [1]. Income inequality is rarely considered as a possible determinant of health levels [2].

Regarding income distribution and inequities, there is great variability in the literature, and the few studies that included such variables, such as the Gini Index, did not find significant correlations with the health level [2]. Some authors have provided evidence that it does not influence health outcomes and it can be considered a mere statistical artifact, simply not sufficiently controlled [3–5]. On the other hand, others have demonstrated that its effect is indirect, with poverty and inequity being able to modify the effect of per capita GDP in achieving health outcomes [6].

The main studies that indicated a consistent association between inequities and health status were carried out by Wilkinson [7], McIsaac and Wilkinson [8], and De Vogli et al. [9]. Some causal pathways between inequities and the level of health have been suggested: socioeconomic factors, such as living and working conditions, housing, education, food, pollution, insecurity; psychosocial factors, such as psychological stress and risk behaviors, such as excessive consumption of alcohol, tobacco and inequalities in access to health services [2, 10]. The proportion of poverty and limited social policies for the health and education sectors have also been highlighted as important causal pathways [11].

In their literature review on income inequity and health levels, Wilkinson and Pickett [12] disclosed that, in 70% of the 168 analyzed studies, the health of populations shows lower levels in societies with greater income inequality. It is worth mentioning that it is important to consider inequity not only from the point of view of income, but adding other dimensions, which can demonstrate it from the social point of view, in the relative position that each one occupies and from their perception, which varies depending on different economic, sociocultural and historical contexts [13]. Thus, it is essential to avoid emphasizing inequities in only one direction, such as income or rights, or even well-being, since they are spaces of constant dispute in the pursuit of equity [14].

Economic inequality has psychological and somatic consequences. Living in an unequal society changes the way people relate to each other, and even how they see themselves. There are close correlations between social inequality and mortality, infant overmortality, lower life expectancy, higher occurrence of mental illness, obesity, homicide, violence, use of illicit drugs, number of people in prisons, lack of trust in other people, teenage pregnancy and less social mobility, among others. The correlations are high, although causal relationships are complex to be established [10, 15, 16].

Unrest is in the air, and evidence suggests a real epidemic of *social status* anxiety in contemporary society, which leads to a negative narcissism, loneliness and the incapacity to establish affective and long-lasting emotional bonds [17]. The most unequal countries have threefold prevalence rates of mental illnesses, such as anxiety and depression. Self-esteem is low, and the lack of self-control is noticeable. Health inequity generates real suffering for almost the entire population: the described problems affect everyone, not just the most vulnerable individuals. There is an increase in competition, of purely materialistic desires and the social hierarchy is strengthened. The fundamental hypothesis is that inequity deteriorates the whole of society and not just the marginalized groups. The population is less willing to help other people, mainly the elderly, to receive immigrants and refugees, and to spend resources on sick individuals [10, 18].

Moreover, there are lower levels of social participation of the most vulnerable part of the population when the level of inequity is high, both from a civil and social point of view, isolating the poorest [19]. In this case, in societies with greater inequity, the relationship between income and social participation is narrowed. Furthermore, countries with high degrees of social inequity have eroded the social cohesion, a real *social corrosion*, with lower levels of social and political participation by civil society, with insufficient levels of social protection and a greater degree of distrust. It is a vicious circle, in which institutions are losing the ability to meet the needs of the most vulnerable groups, which participate less and less in politics [20, 21].

Another relevant point is the timing of effects of inequity in population health: it is known that its effect is not an immediate one. According to Zheng [22], the sufficient time would comprise a five-year period, with a peak at 7 years and decreasing after 12 years.

More egalitarian societies have better levels of health and longevity, according to some authors [23, 24], but some public authorities remain suspicious of this hypothesis, which happens to be most inconvenient to the interests of financial capital movement, and tend to make decisions based on studies that deny this relationship [25], legitimizing the interests of the hegemonic classes.

However, many methodological and empirical criticisms followed the presented evidence that associated income inequality to health level outcomes. Beckfield [26] reproduced previous studies with additional data and detected that the previous associations, which remained in the least squares regressions, disappeared when using the fixed effects models. Mellor and Milyo [27] also demonstrated that, with the inclusion of a series of controls in their analysis, the relationships between income inequality and health levels disappeared. Gerdtham and Johannesson [28] found evidence of the association between income and health levels but did not confirm their relationship with income inequality.

Lynch et al. [24] and Lynch et al. [29] demonstrated that only a few studies in the United States showed an association between inequity and health levels, concluding that the evidence is inconsistent and insufficient. Deaton [25] also stated in his analysis that inequity itself is not a determinant of health levels.

The aim of this article is to evaluate the role of health equity in determining health outcomes, in an international comparative analysis of the effectiveness and efficiency of health systems, contributing to this debate.

## Method

We selected *life expectancy at birth* and *infant mortality* as our main effect variables, for the whole set of analysis. We also evaluated the World Happiness Index, mortality from preventable causes and disability-adjusted life years for chronic diseases, but only in the fixed effects panel regression model. We assessed the following dimensions, permeated by health equity: physical and financial resources, intermediate outcomes of health production (access, coverage and prevention variables) and environmental, demographic, socioeconomic, governance and health risk variables. These dimensions were selected because they allow assessing the efficiency of physical and financial resources upon health levels, health care productivity (resources and health production) and effectiveness, relating health production to outcomes [30]. Table 1 shows the variables used in this study.

**Table 1:** Variables used, according to the stage of the health production process, data sources and the analyzed period

Variables (specifications):	Period 1(t)	Period 2(t + 1)
**INPUT**		
**FINANCIAL RESOURCES**		
**HEALTH EXPENSES - AGGREGATE MEASURES**		
Health expenditure as % of GDP	2010 (WHO)	2014 (WHO)
Total health expenditure per capita PPP (purchasing power parity)	2010 (WHO)	2014 (WHO)
**HEALTH EXPENDITURE - FUNDING SOURCES**		
Public, private and out-of-pocket spending as % of health spending	2010 (WHO)	2014 (WHO)
Public spending on health as % of total government spending	2010 (WHO)	2014 (WHO)
External source of health expenditure (% current health expenditure)	2010 (WHO)	2014 (WHO)
**HEALTH EXPENSES - FINANCING ARRANGEMENTS**		
Government financing arrangements as % of current health expenditures	2010 (NHA)	2015 (NHA)
Social health insurance as % of current health expenditure	2010 (NHA)	2015 (NHA)
**RESOURCES (HUMAN, MATERIALS, TECHNOLOGICAL AND OF GOVERNANCE)**		
Density of health professionals (per 1000 inhabitants)	2010 (WHO)	2014 (WHO)
Density of hospital beds (per 10,000 inhabitants)	2005 (WHO)	2010 (WHO)
Density of hospitals and health centers (per 100,000 inhabitants)	2010 (WHO)	2014 (WHO)
Equipment: (Tomography and Magnetic Resonance machines per 1000,000 inhabitants)	2010 (WHO)	2014 (WHO)
National strategy for the prevention of chronic non-communicable diseases	2010 (WHO)	2014 (WHO)
**OUTPUT**		
**ACCESS AND COVERAGE**		
Treatment coverage for tuberculosis	2010 (WHO)	2015 (WHO)
Medical care seeking for pneumonia symptoms	2010 (WHO)	2015 (WHO)
Treatment coverage for diarrhea	2010 (UNICEF)	2015(UNICEF)
Births performed by skilled health personnel	2010 (WHO)	2014 (WHO)
**PREVENTION**		
Tuberculosis incidence	2010 (WHO)	2014 (WHO)
HIV incidence	2010 (WHO)	2014 (WHO)
Vaccination	2010 (WHO)	2014 (WHO)
**OUTCOME**		
World Happiness Index	2012 (UNSDSN)	2017 (UNSDSN)
DALY – disability-adjusted life years	2010 (WHO)	2015 (WHO)
**Life expectancy at birth**	**2010 (WHO)**	**2015 (WHO)**
**Infant mortality**	**2010 (WHO)**	**2015 (WHO)**
Probability of death from preventable causes (cardiovascular disease, cancer, diabetes and chronic respiratory diseases) between 30 and 70 years old	2010 (WHO)	2015 (WHO)
**INTERSECTORAL VARIABLES (throughput or cross-sectional)**		
**DEMOGRAPHIC**		
Population density	2010–14 (CIA)	–
**SOCIOECONOMIC**		
Gini Index	2010 (PNUD)	2015 (PNUD)
Richest percentile (!%)	2010 (WID)	2015 (WID)
Education Index (component of the HDI)	2010 (PNUD)	2015 (PNUD)
**GOVERNANCE**		
General inequity - HDI losses (all dimensions) and Gender Inequality	2010 (PNUD)	2015 (PNUD)
Unemployment	2010 (PNUD)	2015 (PNUD)
Corruption Perception Index	2012 (TI)	2016 (TI)
Political Regime	2013 (Polity IV)	–
KOF Globalization Index, financial capital economic component	2010 (SEI)	2015 (SEI)
Government Effectiveness, Voice and Accountability and Rule of Law *	2012 (IGBM WB)	2017 (IGBM WB)
**RISK FACTORS (ENVIRONMENTAL, CHRONIC DISEASES, INJURIES)**		
Basic and safe sanitation -% population	2000 (WHO)	2015 (WHO)
Obesity (prevalence; < 5 years)	2008–12 (WHO)	2015 (WHO)
Prevalence of hyperglycemia / diabetes	2010 (WHO)	2014 (WHO)
Prevalence of hypertension	2010 (WHO)	2015 (WHO)
Malnutrition (prevalence; < 5 years)	2008–12 (WHO)	2015 (WHO)
Alcohol (consumption per capita, over 15 years)	2010 (WHO)	2015 (WHO)
Tobacco (prevalence of tobacco use; over 15 years)	2010 (WHO)	2015 (WHO)
Fatal and non-fatal occupational accidents / injury rates [SDG 8.8.1]	2010 (UN)	2015(UN)

Data sources: *WHO* World Health Organization; *SDG* Sustainable Development Goals; *WB* World Bank; *CIA* Central Intelligence Agency; *TI* Transparency International - The Global Anti-Corruption Coalition; *Polity IV* Individual Country Regime Trends; *SEI* Swiss Economic Institute; *UNDP* United Nations Development Program; *NHA* National Health Accounts; *UNICEF* United Nations Children’s Fund; *UNSDSN* United Nations Sustainable Development Solutions Network; *WGI* Worldwide Governance Indicators; *UN* United Nations and *WID* World Inequality Database.

It is important to emphasize that the variable selected to measure inequity was as comprehensive and complex as possible, as it adjusts the Human Development Index (HDI) score in relation to the decrease associated with multidimensional inequality, from the economic (income) and social (education and health) points of view. The HDI, adjusted for inequality, is based on the Atkinson index **A = 1– g/μ**, where g is the geometric mean and μ is the arithmetic mean of the distribution, which satisfies subgroup consistency. This property ensures that any differences in the distribution of human development within only a certain group of the society imply differences in the distribution across the entire society [31]. In this context, gender inequity was also tested, encompassing intersectoral dimensions related to reproductive health, empowerment and the labor market and unemployment, from the perspective of its association with social inequity.

From the governance point of view, the selected indicators are related to the traditions and institutions by which authority is exercised in a given country, including the processes by which governments are chosen, monitored and replaced; the government’s capacity to safely formulate and implement policies; and the respect, attributed by citizens and the Government, to the institutions that govern their social and economic interactions. These indicators are constructed through data sources from different origins, either governmental or not, which capture perceptions about these dimensions [32].

We considered a five-year period, with observations ranging from 2010 and 2015 for all countries, whenever data was available for the studied variables. The sociocultural regions used in the study were the ones proposed by Fischer [33], consisting of the following categories: Sub-Saharan African (central, west, east and south), Arabic/North Africa and Mediterranean, Asian (east, south and southeast), Anglo-Saxon, Central American, Germanic, Oceanic, Ottoman, Latin (Romanic) and Slavic. These regions allow countries to be grouped according to their common linguistic, historical and cultural heritage characteristics, with greater diversity than merely geographical regions, better discriminating the differences between groups.

We performed both Fixed Effects (FE) and Data Envelopment Analysis (DEA) techniques, applied respectively for panel regression model and dynamic network model, in addition to the correlation between methods. We also assessed the possibilities of improvement regarding the outcomes and their impact on health, as the potential gain in life-years and the reduction in infant mortality based on the comparison with benchmark countries for efficiency. It is important to highlight that, while the fixed effects model has a double utility, of presenting both the social determinations of health related to effectiveness over time, and to efficiency (residuals); the dynamic and network DEA model, allows to assess efficiency, considering distinct stages of the health production process, dynamically, over the two-period time.

In order to perform this analysis, our concern in relation to sample size matched our need to assess as much countries as possible in our sample, in all regions. Thus, considering that the number of observations ranged from 129 to 374 country-years, and no more than ten parameters were tested simultaneously, we met the criteria for both methods. Green [34] provides a comprehensive overview of the procedures used to determine regression sample sizes, with *N* > 104 + m (where m is the number of independent variables) for testing individual predictors (assuming a medium-sized relationship). For regression equations using six or more predictors, an absolute minimum of 10 participants per predictor variable is appropriate [35]. As for the DEA model, Cooper et al. [36] suggest that in order to have adequate numbers of degrees of freedom (adequate discriminatory power), the sample size (number of decision-making units) should exceed the number of inputs (m) and outputs (s) by several times. More specifically, they suggest a rule of thumb formula that “n” should be greater than max{m*s, 3* (m + s)}.

The fixed effects panel regression model admits the following assumptions: each unit has its own characteristics that may or may not influence the explanatory variables; some unit characteristic may generate a biased or impaired explanatory power for the variables and, therefore, it is necessary to control this effect. The fixed effects model removes these time-invariant characteristics from the explanatory variables, to allow for a net effect analysis. The country specific effect was calculated as the sum of the country fixed-effect plus the residual in the equation, which can suggest inefficiencies at its upper limit when negative, relativizing the untested variables and measurement errors [2]. Another important assumption is that these individual and time-invariant characteristics are unit-specific and do not correlate with each other. Each unit is different and, therefore, the error term and the constant (which captures these individual characteristics) should not be correlated with those of the other units [37]. We evaluated the heteroscedasticity in the residuals of the estimated regressions and the comparison with the random effect regression. Corrections and regressions were performed, when necessary, using the generalized least squares method. *Stata SE 10.1* software was used for the analysis of the fixed effects regression and its residuals.

The equation shown below was used to obtain the final models:

$$ Yit=\beta o+{\beta}_1{\chi}_{1, it},+\cdots +{\beta}_k{\chi}_{k, it}+{y}_2{\varepsilon}_2+\cdots {y}_n{\varepsilon}_n+{\delta}_2{T}_2+\cdot \cdot {\delta}_t{T}_t+{\mu}_it $$

Where:

–Y_it_ is the dependent variable (DV) where i = unit and t = time.

–*X*_k,it_ represents the independent variables (IV),

–*β*_k_ is the coefficient for IVs,

–*u*_it_ i is the error term,

–ɛ_n_ is the “n” unit.

–γ_2_ is the coefficient for the units.

–T_t_ is time.

–δ _t_ is the time-related coefficient.

For each effect variable, the bivariate association with the independent variables, in addition to the multivariate – by dimension and as a whole, were tested using the fixed effects model. The dimensions comprise: financial and physical resources (efficiency); intermediate health outcomes (effectiveness) and the intersectoral variables related to governance, health, environmental and work risk. Hausman tests (comparison with random effects regression) and heteroscedasticity (constancy of variance, if positive, using a robust standard error technique) were performed. Subsequently, we calculated the country-specific effects, as the sum of fixed effects and residuals. For the final model, we gathered the potential efficiency gains, according to the comparison with the most efficient country (benchmarking).

In the network DEA models, the interconnection between activities is an essential characteristic, with the analysis carried out in multiple stages, seeking to overcome the restrictions of classic and static models and to resemble the characteristics of actual systems [38]. Thus, for a decision-making unit (DMU) to be considered efficient, it must be efficient at all stages of its production process; this technique allows detecting non-radial inefficiencies. In this type of approach, intermediate products are produced and consumed in the DMU itself, while the inputs and outputs are exogenous to its internal structure. In addition to the global score, the network model allows to obtain efficiency indices for each stage. Another characteristic is that the Production Possibility Set (PPS) is modeled at each stage, which has its own technological level, with specific reference coefficients [39]. The *Max Dea 8 Ultra* software was selected to perform the network slack analysis.

The analysis was performed by solving the following linear programming problem, considering the slack-based model:

$$ \underset{\lambda; {s}^{-};{s}^{+}}{\min}\rho =\frac{1-\frac{1}{m}.{\sum}_{i=1}^m{s}_i^{-}/{x}_{i0}}{1+\frac{1}{s}.{\sum}_{r=1}^s{s}_r^{+}/{y}_{r0}} $$

Subject to:

x_o_ = Xλ + s^−^ (input gap vector).

y_0_ = Yλ- s^+^ (output gap vector).

λ ≥ 0, s^−^ ≥ 0, s^+^ ≥ 0 (λ is the intensity vector).

The output-oriented model was considered, with *k* stages, with the following equation:

$$ \frac{1}{\tau_0^{\ast }}=\mathcal{\max}\ {\Sigma}_{\mathcal{k}=1}^{\mathcal{K}}{\mathcal{W}}^{\mathcal{k}}\left\llbracket 1+\frac{1}{{\mathcal{r}}^{\mathcal{k}}+{\sum}_{\mathcal{h}\in {\mathcal{F}}_{\mathcal{k}}}{\mathcal{t}}_{\mathcal{k},\kern0.5em \mathcal{h}\Big)}}\right(\frac{\sum_{\mathcal{k}\mathcal{r}=1}^{\mathcal{r}}{\mathcal{s}}_{\mathcal{r}0}^{\mathcal{k}+}}{{\mathcal{y}}_{\mathcal{r}0}^{\mathcal{k}}}+\frac{\Sigma_{\mathcal{h}\in {\mathcal{F}}_{\mathcal{k}}{\mathcal{s}}_{\mathcal{h}0}^{\left(\mathcal{k},\mathcal{h}\right)+}}}{{\mathcal{z}}_{\mathcal{h}0}^{\left(k,\mathcal{h}\right)}}\Big\rrbracket $$

Subject to:

$$ {{\mathrm{z}}_{\mathrm{ho}}}^{\left(\mathrm{k},\mathrm{h}\right)}={\mathrm{Z}}^{\left(\mathrm{k},\mathrm{h}\right)}\ {\lambda}^{\mathrm{k}}-{{\mathrm{s}}_{\mathrm{o}}}^{\left(\mathrm{k},\mathrm{h}\right)+} $$

$$ {\mathrm{Z}}^{\left(\mathrm{k},\mathrm{h}\right)}\ {\lambda}^{\mathrm{h}}={\mathrm{Z}}^{\left(\mathrm{k},\mathrm{h}\right)}\ {\lambda}^{\mathrm{k}} $$

$$ {{\mathrm{s}}_{\mathrm{o}}}^{\left(\mathrm{k},\mathrm{h}\right)+}\ge 0 $$

where, w^k^ is the relative weight of each region; F_k_ is the set of stages with links (k, h); ∑^K^_k = 1_ w^k^ = 1; w^k^ ≥ 0; s^k+^ are the output slack vectors; r^k^ is the number of outputs in stage k; t _(k, h)_ is the number of products in the link between stages k and h; s_ho_^(k, h)+^ are the link slack vectors and z deals with intermediate products.

For life expectancy at birth (*n* = 161), we evaluated health expenditure per capita (with purchasing power parity - PPP) and inequity variables, measured in relation to losses in the HDI in its several dimensions, as well as tuberculosis treatment and HIV incidence, as intermediate products, in health production.

For infant mortality (*n* = 188), the following variables were used: per capita health expenditure (with purchasing power parity - PPP), direct disbursement, and as intermediate variables: education (HDI), care seeking for diarrhea in children under 5 years of age, births attended by skilled health personnel and the incidence of HIV.

Correlations (Spearman’s non-parametric correlation test) were tested between methods, in order to verify the impact on health, both from the point of view of potential years gained in life expectancy at birth and the reduction in infant mortality rates.

## Results

Table 2 presents the results obtained through bivariate analysis in the fixed effects panel model for different dimensions and health level variables. The financial health care resources were more statistically significant than the physical resources. The health coverage dimension, in health production, was significant in determining results for the set of countries.

**Table 2:** Results of the bivariate analysis, fixed effects model, for the variables life expectancy and infant mortality

Dimension	Variable	LEB* coef	LEB* p	IM** coef	IM** p
Financial resources	Current per capita health expenditure (PPP)	**0.0013**	**0.002**	**−0.0021**	**0.040**
	Health expenditure (% GDP)	−0.0818	0.571	**−0.5409**	**0.098**
	Health expenditure - external sources	−0.0441	0.191	−0.0203	0.794
	Health expenditure (% government expenditure)	0.0513	0.490	−0.2023	0.231
	Taxes (% current health spending)	−0.0057	0.830	0.0288	0.635
	Compulsory contributions (social security)	**0.0980**	**0.017**	**−0.2157**	**0.021**
	Public/mandatory spending	0.0290	0.231	−0.0503	0.362
	Voluntary contributions	−0.0263	0.468	−0.0859	0.299
	Private health insurance	**0.1104**	**0.075**	−0.2206	0.119
	Direct disbursement (OOP)	−0.0263	0.308	**0.1531**	**0.009**
	Private spending	−0.0331	0.16	**0.0919**	**0.086**
Physical Resources	Density of physicians	**0.1070**	**0.059**	**−0.2250**	**0.071**
	Density of nurses/midwives	0.0211	0.439	− 0.0470	0.424
	Density of hospital beds	**−0.1012**	**0.080**	0.0791	0.417
	Density of Health Units	−0.0051	0.811	−0.0155	0.802
	Density of Health Centers	−0.0086	0.940	0.1792	0.469
	Density of Hospitals	−0.3136	0.378	0.2453	0.536
	Density of Magnetic Resonance Imaging equipment	0.0645	0.531	−0.0688	0.809
	Density of Computed Tomography equipment	0.0794	0.498	−0.0726	0.772
	National CNCD prevention strategy	**0.6594**	**0.067**	−1.2000	0,133
Coverage	Tuberculosis Treatment	**3.8289**	**0.046**	**−9.8135**	**0.002**
	Care seeking - Pneumonia	**0.1399**	**0,001**	**−0.2224**	**0.025**
	Care seeking - Diarrhea	**0.0623**	**0.062**	**−0.2851**	**< 0.001**
	Skilled personnel - births	**0.0739**	**< 0.001**	**−0.2717**	**< 0.001**
Prevention	Tuberculosis incidence	**−0.0149**	**< 0.001**	**0.0375**	**< 0.001**
	HIV incidence	**−2.8490**	**< 0.001**	**6.8174**	**< 0.001**
	Complete immunization (< 1 year)	0.0264	0.632	**−0.2121**	**0.076**
Governance	Demographic density	**0.0042**	**0.064**	−0.0067	0.183
	Gini index	**−0.1168**	**0.081**	0.0979	0.587
	Percentile of the richest (1%)	10.1313	0.437	0.7650	0.960
	HDI Education	**39.2567**	**< 0.001**	**− 93.2400**	**< 0.001**
	HDI	**64.3705**	**< 0.001**	**− 154.4554**	**< 0.001**
	General inequity - HDI losses	**−0.2453**	**< 0.001**	**0.7767**	**< 0.001**
	Gender inequity	−0.4598	0.385	**2.1133**	**0.051**
	Unemployment	**−0.2446**	**0.003**	0.2789	0.132
	Political Regime	0.0091	0.500	−0.0315	0.301
	Voice and Accountability	**1.3940**	**0.099**	**−4.0541**	**0.030**
	Government Effectiveness	0.7931	0.289	−1.1107	0.498
	Rule of Law	**1.9783**	**0.009**	**−4.0573**	**0.017**
	Corruption Perception Index	**0.1345**	**0.001**	**−0.4388**	**< 0.001**
	Financial globalization	0.0271	0.397	**−0.1185**	**0.096**
Risk factors	Safe sanitation	**0.1209**	**< 0.001**	**−0.2573**	**< 0.001**
	Basic sanitation	**0.2433**	**< 0.001**	**−0.7931**	**< 0.001**
	Obesity	**0.5446**	**< 0.001**	**− 1.2582**	**< 0.001**
	Diabetes	**1.4695**	**< 0.001**	**−4.4496**	**< 0.001**
	Hypertension	**−0.6278**	**< 0.001**	**0.9702**	**0.004**
	Malnutrition	**−0.2167**	**< 0.001**	**0.7989**	**< 0.001**
	Alcohol	**0.4031**	**< 0.001**	**−1.1657**	**< 0.001**
	Tobacco	−0.0749	0.256	−0.0366	0.786
	Occupational accident injuries	**−0.0004**	**0.094**	0.0003	0.475
	Fatal occupational injuries	**−0.1707**	**0.009**	**0.3098**	**0.011**

* *LEB* Life expectancy at birth ** *IM* Infant mortality; *OOP* Out-of-pocket expenses; *CNCD* Chronic non-communicable diseases

Based on the bivariate analysis, the models for the different dimensions and also the general ones were tested, selecting the relevant variables from the analyzed dimensions. Table 3 aggregates the results, showing that the inequity variable, expressed as the losses in the results of the human development index, due to inequalities in the dimensions of education, health and income, was significant for both effect variables, remaining in the final model of the variable *life expectancy at birth*, together with health expenditure per capita. In contrast, *infant mortality* comprehended education as essential for its reduction, in association with variables of the health production dimension, such as the health care seeking due to diarrhea in children under 5 years old, births performed by skilled personnel and the reduction in HIV incidence. Both models showed high values of R^2^, close to 70%. An interesting point is that from the perspective of financial resources, direct disbursements carried out by the population were harmful to infant mortality, while social or private insurance proved to be beneficial to *life expectancy at birth*. Regarding health production, treatment for tuberculosis and care seeking due to pneumonia were decisive for the increase in life expectancy. In the health and environmental risk dimension, malnutrition, hypertension, fatal occupational injuries and lack of basic sanitation have shown to be relevant variables for both effects, with obesity, alcohol consumption and the prevalence of diabetes mellitus showing an inverse association with infant mortality. Regarding governance, it is worth mentioning that *Rule of Law* appears as a determinant of *life expectancy at birth*. This variable captures the perception of the extent to which agents trust the rules of society, particularly the quality of contracts enforcement and property rights, the police and the courts, as well as the likelihood of crimes and violence. Among the dimensions assessed (physical and financial resources; health production, governance and environmental risks), governance was the one with the highest R^2^ values, followed by environmental risks, health production and, finally, financial resources. Other variables were significant only in the bivariate analysis, not remaining in the final models, such as voice and accountability, unemployment, gender inequity, Gini index, financial globalization and perception of corruption.

**Table 3:** Final regression models for the selected health outcomes, according to partial and general models (fixed effect models)

Model/Variable	Life Expectancy at Birth (*n* = 161)	Infant mortality (*n* = 188)
Physical / financial resources	Per capita health expenditure Compulsory social insurance Private health insurance (*R*^*2*^ = 38.79%; *p* < 0.0001; *n* = 186)	Per capita health expenditure (−) Direct disbursements (OOP expenses) (^*R2*^ = 32.54%; *p* < 0.0001; *n* = 188)
Health Production	Tuberculosis treatment Pneumonia (care seeking) * HIV incidence (−) (*R*^*2*^ = 44.29%; *p* < 0.0001; *n* = 99)	Diarrhea (care seeking) - Birth performed by skilled personnel (−) HIV incidence (*R*^*2*^ = 36.42%; *p* < 0.0001; *n* = 87)
Governance environment	Education Inequity (−) Rule of law (*R*^*2*^ = 70.51%; *p* < 0.0001; *n* = 162)	Education (−) Inequity (*R*^*2*^ = 75.59%; p < 0.0001; n = 162)
Health and environmental risks	Basic sanitation Malnutrition (−) (*R*^*2*^ = 64.55%; *p* < 0.0001; *n* = 139)	Basic sanitation (−) Diabetes Mellitus prevalence (−) Malnutrition (*R*^*2*^ = 43.91%; *p* < 0.0001; *n* = 139)
General - all dimensions	Per capita health expenditure **Inequity** (−) (*R*^*2*^ = 71.31%; *p* < 0.0001); *n* = 161	Diarrhea (care seeking) - Birth performed by skilled personnel (−) HIV incidence **Education** (−) (*R2* = 67.19%; *p* < 0.0001; *n* = 87)

*removed, after robust standard error

The final FE model regression equations obtained were:

$$ Life\kern0.17em Expectancy\; at\; Birth=74.4539+0.0009556\ast per\; capita\kern0.17em health\kern0.17em expenditure-0.5238\ast social\kern0.17em inequity $$

$$ Infant\kern0.17em Mortality=98.2248-90.0532\ast level\kern0.17em of\kern0.17em education-0.0900\ast care\kern0.17em seeking\kern0.17em for\kern0.17em diarrhea\kern0.17em in\kern0.17em children\kern0.17em under\kern0.17em five-0.1580\ast birth\kern0.17em performed\; by\; skilled\kern0.17em personnal+3.7875\ast HIV\; incidence $$

We also studied associations with other effect variables, such as the World Happiness Index, mortality from preventable causes and disability-adjusted life years, specifically for chronic non-communicable diseases (CNCD).

Regarding the *World Happiness Index*, the following variables remained in the final model (*R*^*2*^ = 67.19%; *p* < 0.0001): tuberculosis incidence, unemployment (negative sign) and Government Effectiveness. This variable captures the perceptions about the quality of public and civil services and the degree of its independence from political pressure, the quality of formulation and implementation of public policies and the government’s credibility regarding its commitment to these policies. It is important to note that private insurance, in the health resources dimension, showed an opposite sign to the health outcome. In the case of tuberculosis incidence, it is possible to assume that health professionals are attentive to the correct diagnosis and appropriate treatment, considering that the detection has increased more than new cases.

Respecting *mortality from preventable causes*, care seeking due to diarrhea in children under 5 years of age and inequity remained in the final model. In relation to the dimensions, it is worth mentioning that the density of physicians and the existence of a strategy to reduce CNCD have a protective effect, in addition to births performed by skilled health professionals. As for the environmental variables, gender inequity and unemployment appear to increase mortality, while education and corruption control favor its prevention (*R*^*2*^ = 28.12%; *p* < 0.0001).

Concerning the *disability-adjusted life years* (DALYs) for CNCD, proportionally to the total of DALYs, the final model (*R*^*2*^ = 85.90%; *p* < 0.0001) consisted of: births performed by skilled health professionals; Education and Rule of Law, with a positive sign, and HIV incidence and Gini index, with a negative sign.

**Fig. 1:**
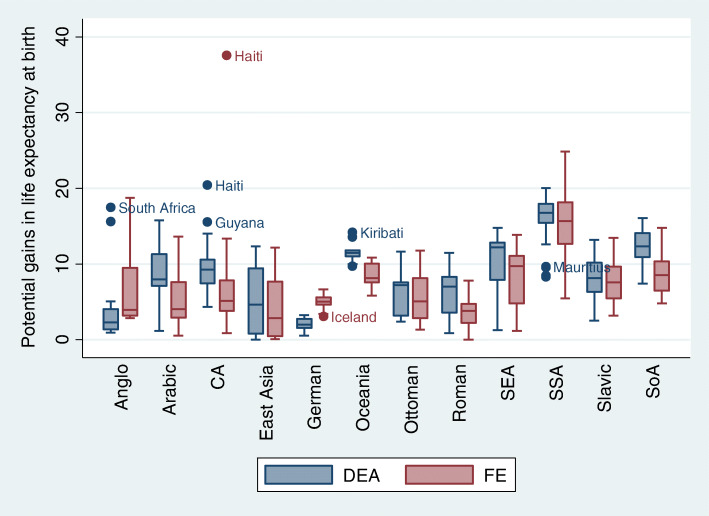
Distribution of potential years gained in life expectancy at birth, by sociocultural regions and applied methods (FE and DEA)

Figure 1, below, shows the potential years gained in *life expectancy at birth* according to sociocultural regions. We observed that, in most sociocultural regions, the distributions obtained according to the DEA method showed higher values than in the FE model, including more countries displaying extreme values (outliers). Interestingly, in the Anglo-Saxon and Germanic regions and, to a lesser extent, in the Slavic region, results were superior using the FE model. The following countries revealed greatest potential for years gained: South Africa, Haiti, Guyana and Kiribati, and the reference countries are Iceland and Mauritius, as they stand out as best performers.

**Fig. 2:**
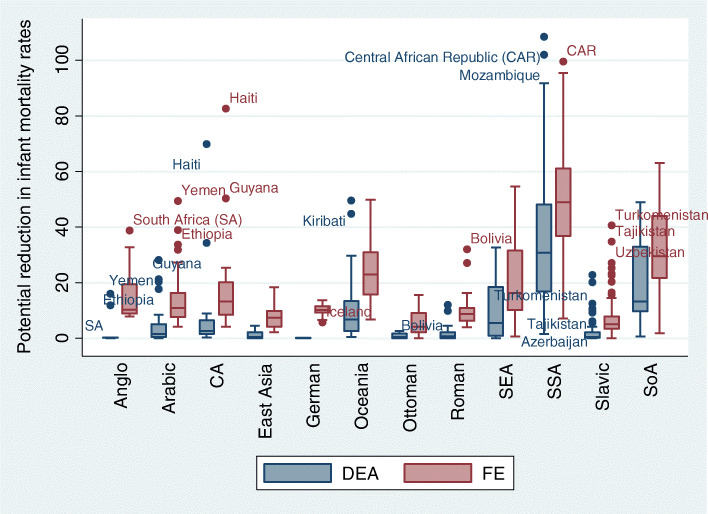
Distribution of the potential reduction in infant mortality rates, by sociocultural regions and applied methods (FE and DEA)

Figure 2 shows the potential reductions in *infant mortality* rates by sociocultural regions. Contrary to life expectancy, we depict that in the case of infant mortality, the distributions showed higher values in the FE model than in the DEA model. Additional countries stand out as outliers, with greater potential for reducing infant mortality rates in Sub-Saharan Africa (Central African Republic and Mozambique), in the Slavic region (Turkmenistan, Tajikistan, Azerbaijan and Uzbekistan) and in the Latin region (Bolivia). Iceland, in the opposite direction, remains a benchmark country.

**Fig. 3:**
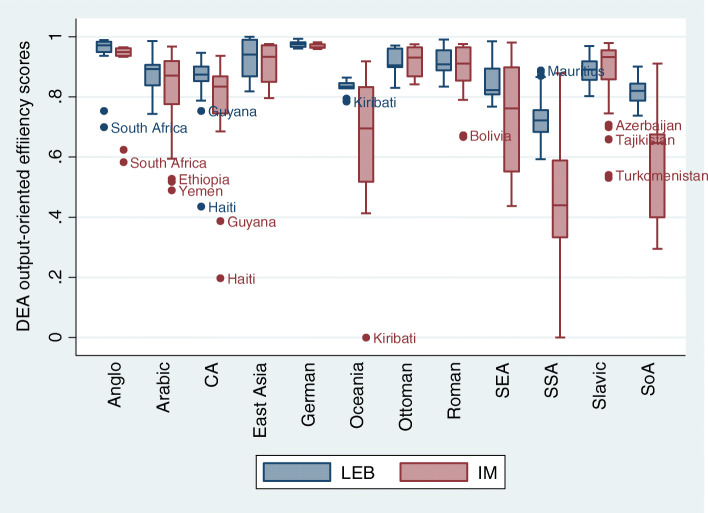
Distribution of efficiency scores oriented to potential years gained in life expectancy at birth and the reduction in infant mortality rates, by sociocultural regions

Figure 3 shows the distribution of DEA efficiency scores obtained for both effect variables. We note that *life expectancy at birth* efficiency scores distributions are higher than those of *infant mortality rates*, except for the Slavic region. The Germanic, Latin and Ottoman regions show similar distributions for health outcomes.

The supplementary graphs (Additional files 1, 2 and 3) compare efficiency scores between the fixed effects model (FE) and the Data Envelopment Analysis (DEA) and show the potential gains in life expectancy and potential reductions in infant mortality, by sociocultural region, benchmarked by best performing countries. Thus, it is possible to compare the absolute values obtained by FE and DEA techniques for life expectancy (Additional file 1) and infant mortality (Additional file 2), as well as to observe the relative proportions of efficiency, according to the DEA for both variables (Additional file 3). For example, South Africa showed an efficiency close to 72.62%, indicating the possibility of increasing life expectancy by 27.8%, representing a gain of 16.53 years by DEA (0.2738*60.65) and 15.96 years by the FE model. In the case of the United States, it is noteworthy that the FE model (10.03 years) returns a potential gain in life expectancy well above that obtained by DEA (4.56 years), since effectiveness is measured beyond efficiency in that method.

Overall, the efficiency averages were higher for life expectancy, with smaller variations than infant mortality. The difference between the efficiency indices reached 30 percentage points between the two variables in some locations. Major inefficiencies were found for infant mortality, mainly in the regions of Central America (Haiti), Oceania (Kiribati), Southeast (Laos and East Timor) and South Asia (Pakistan and Afghanistan) and Sub-Saharan Africa (Sierra Leone, Mozambique, Lesotho and Central African Republic).

We gather that, in all sociocultural regions, countries that have the lowest efficiency rates and, therefore, the greatest potential for improvement, are those with the highest level of poverty and or inequities. The existence of conflicts, wars, genocides, predatory colonization, geographic isolation and environmental disasters enhance these results for both methods employed. For instance, in the Anglo-Saxon region, the USA and South Africa represent the main niches of potential gains in life expectancy and reductions in infant mortality rates. Ethiopia and Yemen, in the Arabic/North African region; Haiti and Guyana, in Central America; Mongolia and China in East Asia; Denmark and Germany, in the German region (including the Netherlands and Switzerland, in the case of infant mortality); Kiribati in Oceania; Moldova and Hungary, in the Ottoman region (as well as Turkey, for infant mortality); Bolivia, Venezuela, Romania and Brazil in the Romanic/Latin region; Laos and Myanmar (in addition to East Timor, for infant mortality), in Southeast Asia; Russia and Central Asian countries, in the Slavic region; Afghanistan, Pakistan and India in South Asia. Finally, in the sub-Saharan Africa, countries in the south, central and western parts, such as Sierra Leone, Lesotho, Central African Republic, Ivory Coast and Chad, predominate.

However, efficiency levels vary greatly between regions: while the Germanic region starts off well above 95%, both for life expectancy at birth and for infant mortality rates, Central America and Sub-Saharan Africa regions show much lower rates, of 60% for life expectancy at birth (up to 24 years of life gained) and 30%, for infant mortality rates (reduction of more than 60 points). In South and Southeast Asia, infant mortality efficiency scores also exhibit reduced values, of about 40% (reduction of 49–57 points), while the Arabic region reaches no more than 50% (reduction of 28 points).

We also tested the correlations between the results obtained with both techniques, for the potential gains in life expectancy or reductions in infant mortality rates. We report strong correlations for *life expectancy* (**− 0.9383**), ranging from − 0.9425 in 2010 to − 0.9336 in 2015, and for *infant mortality* (**− 0.9618)**, ranging from − 0.9718 in 2010 to − 0.9618 in 2015.

**Fig. 4:**
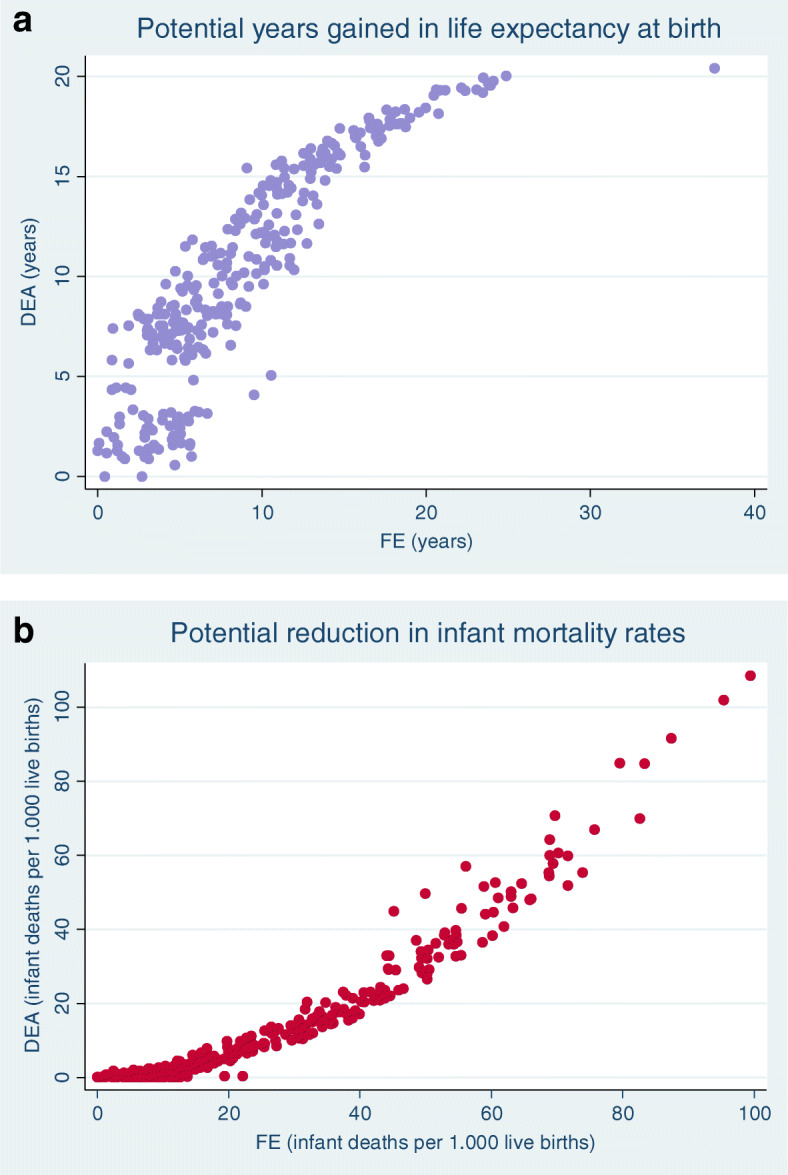
a Correspondence of results of potential gains in life expectancy, DEA and FE methods. b Correspondence of the results of potential reduction in infant mortality rates, DEA and FE methods

The graphs below (Fig. 4a and b) show the correlation between the two methods, for the general dimensions.

## Discussion

We demonstrated the importance of including inequity in a more comprehensive way, permeating the social dimension and not exclusively limited to income. In the case of infant mortality, education revealed to be more relevant, along with health production, even though inequity was highly significant in the governance dimension alone. Avendano [40] demonstrated that infant mortality was not explained by income inequality, most likely because the majority of countries with health policies favorable to its reduction acted intersectorally, presenting more homogeneous income distributions.

Countries with a socialist past or that showed an earlier and long lasting model of the Welfare State in the capitalist system, seem to show greater resilience of their health levels in periods of adversity, providing additional protection in relation to the economic crises of capitalism, far beyond their wealth [41–43]. In this sense, we observed that the Slavic and Ottoman regions show differential levels of efficiency, even in poorer locations, such as Central Asian countries. The Germanic region, in addition to some countries in the Anglo-Saxon (Canada and United Kingdom) and Latin (France) regions have high levels of efficiency, consistent with more extended social protection. This residual effect, from the socio-historical point of view, manifests itself more as life expectancy than infant mortality, considering the cumulative effect observed in the first one, although prioritizing education also has an effect on the latter. The most affected countries, in this sense, are those that have undergone a more accelerated globalization process, without the prior consolidation of social protection mechanisms, such as the countries of Central America and Asia (south and southeast).

It is noteworthy that those countries belonging to the BRICS (Brazil, Russia, India, China and South Africa) or MIST (Mexico, Indonesia, South Korea and Turkey) have results far below what would be expected from previous predictions, largely due to inequities and the huge income and political power concentrations among the dominant elites, which even hinder economic growth. South Africa stands out negatively in this aspect, in our analysis (60 to 73%), whereas South Korea shows high efficiency results (97 to 98%). The extreme inequalities observed in Brazil, India and South Africa, as well as in Middle East countries, hinder social mobility, access to quality education and productivity. Their causes vary widely, but issues deeply rooted in societies, such as racism and racial segregation, the institutionalization of a caste system, excessive deregulation and the excessive exploitation of natural resources, are important determinants for these results [44].

There is a strong correlation between the statistical methods applied, in agreement with Joumard [2], demonstrating that the possibility of gain in life expectancy or infant mortality reduction should be undertaken by public authorities, from a perspective of expanding health resources, which should not be wasted, and where equity should be given its deserved importance, ideally not only income related but also socially concerned [13]. In the case of infant mortality, it is important to prioritize education and the aspects related to health production, such as births performed by skilled professionals, care seeking due to diarrhea in children under 5 years and the reduction in HIV incidence. Income inequality remains in the background in this matter, with equitable access to education and health being more important. On the other hand, direct spending on health (out-of-pocket) has a deleterious association with infant mortality.

Most studies that employ DEA develop their analysis in two stages; thus, after the efficiency operationalization, regressions are performed to evaluate the factors that influence inefficiency. In these evaluations, the Gini coefficient is usually tested; therefore, only income inequality is more frequently analyzed, sometimes remaining as a significant variable, together with per capita income [45, 46]. These authors found an inverse association between the proportion of public funding and health systems efficiency, which leads to the discussion about the relationship between the public-private mix (differences in the public and private funding ratios) and health efficiency, which was not significant in several studies [2]. Berger and Messer [47] found an association between the proportion of health spending, healthy lifestyles and a higher level of education with reduced mortality. Income inequalities were not significantly associated; additionally, the increase in the proportion of public spending was associated with higher mortality, as opposed to outpatient coverage by private insurance.

Or [48], on the other hand, showed an association between a high proportion of public funding and lower levels of infant mortality; however, it did not influence life expectancy at age 65. He demonstrated that a high number of per capita physicians is associated with lower rates of premature mortality, perinatal and infant mortality, as well as increased life expectancy at age 65 and a lower incidence of heart disease. Verhoeven et al. [49]. also demonstrated the association between higher levels of efficiency with a higher proportion of immunization and medical consultations. Conversely, they demonstrated that most inefficiencies should be attributed to the lack of cost-effectiveness in the acquisition of medical resources, mainly medication, in addition to high expenditures on the salaries of health professionals, in line with the neoclassical economic theory, prioritizing the relationship between health production and health levels, concealing the financial capital movement [50].

In our analysis, the density of physicians was a significant variable only in the bivariate analysis. Overall, financial resources remained with greater strength in the analyzed models, with more rigorous results in comparison with physical resources and in agreement with most of the analyzed articles [2]. In the analysis of mortality from preventable causes, using the fixed effects model, the density of physicians was a relevant variable regarding resources, although it did not remain in the final model where the care seeking due to diarrhea in children under 5 years and inequity prevailed.

Differently from many authors, Elola et al. [51], considered that per capita health expenditure explains a greater proportion of the variance in infant mortality than per capita GDP. Health expenditures are inversely correlated with premature mortality in women and positively correlated with life expectancy in women. Contrastingly, among the assessed European countries, income distribution was not an explanatory variable for the health level. Countries with universal health systems showed greater efficiency in reducing infant mortality, compared to countries with social security. However, no statistical association was found between the health system organization and the health level. More important than the health system organization, the direction towards health financing as a society’s priority, which is sensitive to an equitable distribution, seems to better determine health levels [52, 53].

It is known that a limitation of the DEA methodology is that the efficiency frontier is built based on the comparison between countries; therefore, in our analysis, we tried to include other variables in the model, considering distinct health-production process stages and selecting an output oriented slack model, in order to mediate the construction of this frontier. Dhaoui [54] carried out an efficiency analysis of the countries in North Africa and the Middle East and found that the per capita income did not influence the results, and that the health level did not prevent countries with intermediate results from remaining on the efficiency frontier. The authors found a positive association with private health financing and corruption control. In our analysis, corruption control was protective for the variable mortality from preventable causes, but it did not remain in the final model.

It is important to consider the reasons why the governance variables proposed by the World Bank, political regimes and financial globalization did not remain in the final models. These variables are relevant and often praised by governments; however, in our study, they did not determine health levels. Thus, we realize that governance is important to increase the transparency of decisions, social participation in democratic regimes and citizenship, when fighting for social rights. The great challenge is the search for the proper balance between globalization and the implementation of measures necessary to reduce infant mortality, aimed at reducing inequities in education and health. Corruption prevention can help to increase the effective proportion of health expenditure and reduce inequities caused by inappropriate privileges. However, the final determinant effect of health levels and efficiency is more strongly related to health equity, education, the proportion of health expenditure and health prevention. There is no trade-off between health equity and the increase of the average health level of the population Joumard et al. [55].

In our study, the variable *Government Effectiveness* remained as a significant variable and inversely related to the prevalence of chronic diseases in the governance dimension, when analyzing the proportion of DALYs per chronic diseases. However, it did not remain in the final model, unlike its correlate, the *rule of law*. In the analysis of the world happiness index, it remained in the final model with a positive sign. Lionel [56] analyzed data from 150 countries, according to income, concluding that the emission of carbon dioxide, per capita GDP, the control of corruption, population density, the age ranges of the population and government effectiveness were decisive for health expenditure efficiency. This efficiency was obtained by the DEA, based on per capita health expenditure, considering the results in life expectancy at birth and infant mortality.

It is important to note that the variable *Rule of Law* also remained as relevant in the model for determining life expectancy in the governance dimension. In other words, the capitalist system, based on the construction of the Rule of Law, relies on private property and the implementation of contracts and allows a longer survival, although this survival is linked to a higher proportion of years of life lost due to disability and chronic diseases. However, in the final model, what actually remained as relevant was health expenditure and its distribution, considering social inequities beyond the income.

The variable *voice and accountability* also showed significant results, but only in the partial models of the governance dimension. This variable captures the perception about the extent of citizens’ participation in the choice of government, as well as freedom of expression, association and media freedom. According to Lancee and Werfhorst [19], social participation is modified by inequities in health, with lower participation of vulnerable groups in societies with a high degree of inequity.

Ravangard et al. [57] studied the technical efficiency of the health systems in the organization for economic cooperation, between 2004 and 2010 and found significant associations between per capita GDP and current per capita health expenditure regarding life expectancy and infant and child mortality. No associations were found regarding variables related to physical or environmental resources, such as education and smoking. In our study, we found an association between environmental variables, such as malnutrition, hypertension, fatal occupational injuries and lack of basic sanitation, and both effects, with obesity, alcohol consumption and the prevalence of diabetes mellitus showing an inverse association with infant mortality, showing the contradictions between the scarcity and excess of consumption in capitalism [58]. We understand that these results express a disturbing reduction in social status, because although financial resources are essential, as also demonstrated in our analysis, equally important is how they are distributed, under what perspectives and how they are configured in society. Therefore, Biggs et al. [6] demonstrated that in times of reduction or stabilization of poverty and inequity, the relationship between material living conditions (per capita GDP) positively influenced health levels, both mediated by life expectancy and infant mortality. On the contrary, when there was an increase in poverty and/or inequity, there was only a residual effect between the studied variables.

Pritchett and Filmer [59] demonstrated that the variation in infant mortality between countries was mainly attributed to a set of variables: per capita income and income distribution, education among women, ethnic fragmentation and the predominant religion. Public health expenditure was of little importance. However, these variables are not widely available for international comparisons. Therefore, it was not possible to test the variables ethnicity and religion.

### Limitations

The main limitations of this article are related to the different data sources used for international comparisons, which are not always complete and show some discrepancies in their construction. Many variables are not yet available, especially when considering the construction of historical series. Health policies are also not subject to evaluation in this format, despite generating inequities [60].

The ways social inequities are measured are also quite restricted, as it would be interesting to measure the social status perception, in addition to social position, in the perspective of evaluating social stratification.

The analyzed aggregated data can also hide important relationships within countries, which would be detected at the individual and local levels [6]. However, only comparisons between different countries and sociocultural regions allow us to detect differences that would not be perceived in more homogeneous regions, even with more disaggregated data.

Moreover, any mathematical model tends to reduce the reality. However, aiming to operationalize the elements that we deem relevant to their restoration, we understand that the methods used in this study were the most appropriate ones. Furthermore, the operationalization of the totality of reality is an impossible task and makes it difficult to propose alternatives.

## Conclusion

In this article, we demonstrate that direct disbursement for access to health services is harmful, while education is protective against infant mortality. If, on the other hand, the existence of social or private insurance is beneficial to life expectancy, it did not persist in the final model, which showed equity, together with per capita health expenditure, as determinants of extended survival.

As long as public managers and political leaders remain not sensitized to the distribution of resources, we reiterate, not only from the point of view of income, but also of access to education and health, we will not reach the full potential shown here by the efficiency analysis.

Another important point is that, although international organizations are always stimulating the health efficiency discourse, capitalism produces excesses and waste that do not favor the efficient use of resources [58].

Furthermore, considering the movements of accumulation by exploitation for social reproduction, what currently gives new impetus to capitalism is precisely the accumulation by spoliation [61], transforming previously public niches into possibilities for commodification and privatization. Therefore, health today is seen as an exceptional locus in this sense.

We must ask ourselves how many children will not survive, mainly in peripheral capitalism, while access to health is restricted, in packages or selective forms of primary health care and access to education, while those who survive have their existence narrowed by inequities, aggravated by the plunder exercised by capitalism. Spoliation of all sorts can be perceived, in the most diverse ways, such as the privatization of basic sanitation and the expropriation of natural wealth, while absurd patterns of coexistence between malnutrition and obesity persist, which denote exactly a society of excesses and deprivations, a society with mental and degenerative diseases, with a great burden of suffering, loneliness, inability to maintain affective bonds and praising the new and the loss of memory and subjectivity [62].

As we pondered in our article, the poverty and inequities generated by the existence of conflicts, wars, genocides, predatory colonization, geographic isolation and environmental disasters are exacerbated during crises, as they create servitude and enslavement of vulnerable groups on a global scale, whereas they are seen as opportunities for those who concentrate wealth [63].

The dissociation between the distribution of health outcomes and the overall health level of the population characterizes a disastrous political choice for society, as it is associated with high levels of segregation, disrespect and violence from within. Countries should consider health equity as a priority, adding value to their resources, since health inequities affect the whole of society, with a reduction in social trust and cohesion.

## Supplementary Information

**Additional file 1.** Potential years gained in life expectancy at birth, sociocultural regions: comparison between methods (FE x DEA). This file depicts the amount of potential years gained in life expectancy at birth, calculated with both techniques used, permitting comparisons between countries, within sociocultural regions. Africa has been subdivided further, offering more detailed results.

**Additional file 2.** Potential reduction in infant mortality rates, sociocultural regions: comparison between methods (FE x DEA). This file presents the potential reduction in infant mortality rates, calculated with both techniques used, permitting comparisons between countries, within sociocultural regions. Africa has been subdivided further, offering more detailed results.

**Additional file 3.** DEA output-oriented efficiency scores, sociocultural regions: life expectancy at birth and infant mortality. This file exhibits figures of DEA efficiency scores, permitting comparisons between both outcome variables for countries, within sociocultural regions. Africa has been subdivided further, offering more detailed results.

## Data Availability

The datasets used during the current study are available from the corresponding author upon reasonable request.

## Abbreviations

HDI: Human development index
FE: Fixed effects
DEA: Data envelopment analysis
DALY: Disability adjusted life years
CNCD: Chronic non-communicable diseases
LEB: Life expectancy at birth
IM: Infant mortality

## References

[1] Lynch JW, Davey Smith G, Kaplan GA, House JS (2000). Income inequality and mortality: importance to health of individual income, psychosocial environment, or material conditions. BMJ..

[2] Joumard I, André C, Nicq C, Chatal O (2008). Health status determinants: lifestyle, environment, health care resources and efficiency.

[3] Gravelle H (1998). How much of the relationship between population mortality and unequal distribution of income is a statistical artifact?. BMJ..

[4] Lorgelly PK, Lindley J (2008). What is the relationship between income inequality and health? Evidence from the BHPS. Health Econ.

[5] Mackenbach JP (2002). Income inequality and population health. BMJ..

[6] Biggs B, King L, Basu S, Stuckler D (2010). Is wealthier always healthier? The impact of national income level, inequality, and poverty on public health in Latin America. Soc Sci Med.

[7] Wilkinson RG (1992). Income distribution and life expectancy. BMJ..

[8] McIsaac SJ, Wilkinson RG (1997). Income distribution and cause-specific mortality. Eur J Pub Health.

[9] de Vogli R, Mistry R, Gnesotto R, Cornia GA (2005). Has the relation between income inequality and life expectancy disappeared? Evidence from Italy and top industrialised countries. J Epidemiol Community Health.

[10] Wilkinson RG, Pickett KE (2018). The Inner Level: How More Equal Societies Reduce Stress, Restore Sanity and Improve Everyone’s Well-being.

[11] Whitehead, M, Dahlgren, G. European strategies for tackling social inequalities in health: Levelling up part 2.University of Liverpool: WHO Collaborating Centre for Policy Research on Social Determinants of Health, 2006 (Studies on social and economic determinants of population health, No. 3). http://www.euro.who.int/__data/assets/pdf_file/0018/103824/E89384.pdf

[12] Wilkinson RG, Pickett KE (2006). Income inequality and population health: a review and explanation of the evidence. Soc Sci Med.

[13] Goldthorpe JH (2010). Analysing social inequality: a critique of two recent contributions from economics and epidemiology. Eur Sociol Rev.

[14] Siqueira-Batista R, Schramm FR (2005). A saúde entre a iniquidade e a justiça: contribuições da igualdade complexa de Amartya Sen. Cien Saude Colet.

[15] Charlesworth SJ, Gilfillan P, Wilkinson R (2004). Living inferiority. Br Med Bull.

[16] Wilkinson RG (2004). Why is violence more common where inequality is greater?. Annals N Y Acad Sci.

[17] Caniato AMP, Nascimento MLV (2010). The subjectivity in the consumer society: About the narcissistic suffering in excess and deprivation times. Arq Bras Psicol.

[18] Marmot M (2004). Status syndrome. How your social standing directly affects your health and life expectancy.

[19] Lancee B, Werfhorst HGV (2012). Income inequality and participation: A comparison of 24 European countries. Soc Sci Res.

[20] Daniels N, Kennedy BP, Kawachi I (1999). Why justice is good for our health: the social determinants of health inequalities. Daedalus..

[21] Kawachi I, Kennedy BP (1999). Income inequality and health: pathways and mechanisms. Health Serv Res.

[22] Zheng H (2012). Do people die from income inequality of a decade ago?. Soc Sci Med.

[23] Subramanian SV, Kawachi I (2004). Income inequality and health: what have we learned so far?. Epidemiol Rev.

[24] Lynch JW, DaveySmith G, Harper S (2004). Hillemeier M.is income inequality a determinant of population health? Part2.US national and regional trends in income inequality and age-and cause-specific mortality. Milbank Q.

[25] Deaton A (2003). Health, inequality, and economic development. J Econ Lit.

[26] Beckfield J (2004). Does income inequality harm health? New cross-national evidence. J Health Soc Behav.

[27] Mellor JM, Milyo J (2003). Is exposure to income inequality a public health concern? Lagged effects of income inequality on individual and population health. Health Serv Res.

[28] Gerdtham UG, Johannesson M (2004). Absolute income, relative income, income inequality, and mortality. J Hum Resour.

[29] Lynch JW, Davey Smith G, Harper S, Hillemeier M, Ross N, Kaplan GA (2004). Is income inequality a determinant of population health? Part1. A systematic review. Milbank Q.

[30] Tchouaket EN, Lamarche PA, Goulet L, Contandriopoulos AP. Health care system performance of 27 OECD countries. Int J Health Plann Manag. 2012;27(2) [cerca de 24 p.]. [acessado 2018 out 10] 10.1002/hpm.1110. 10.1002/hpm.1110 22302676

[31] UNDP - United Nations Development Programme. Human Development Data Center. Human Development Report 2020 Technical notes fron UNDP site. Available at: http://hdr.undp.org/en/data

[32] Kaufmann D, Kraay A, Mastruzzi M (2010). The worldwide governance indicators methodology and analytical issues no. 5430.

[33] Fischer JAV (2010). Accounting for Unobserved Country Heterogeneity in Happiness Research: Country Fixed Effects versus Region Fixed Effects [internet].

[34] Green SB (1991). How many subjects does it take to do a regression analysis?. Multivar Behav Res.

[35] Voorhis CRWV, Morgan BL (2007). Understanding power and rules of thumb for determining sample sizes. Tutor Quant Methods Psychol.

[36] Cooper WW, Seiford LM, Tone K (2007). Data envelopment analysis: A comprehensive text with models, applications, references and DEA-solver software.

[37] Woolridge JM (2006). Introdução à econometria: uma abordagem moderna.

[38] Mariz FBAR (2015). Modelos dinâmicos de análise envoltória de dados: revisão da literatura e comparação de modelagens [dissertação].

[39] Cook WD, Zhu J (2014). (Coord.). Data envelopment analysis: A handbook of modeling internal structure and network.

[40] Avendano M (2012). Correlation or causation? Income inequality and infant mortality in fixed effects models in the period 1960–2008 in 34 OECD countries. Soc Sci Med.

[41] Chernichovsky D, Potapchik E, Barnum H, Tulchinsky T (1996). The Russian Health System in Transition: Coping with Old and New Challenges.

[42] Klugman J, Schieber G, Heleniak T, Hon V, Nelson J, Tilly C, Walker L (1998). Health Reform in Russia and Central Asia. Transforming post-communist political economies.

[43] Ruckert A, Labonté R. Health inequalities in the age of austerity: The need for social protection policies. Soc Sci Med. 2017;187 [cerca de 6 p.]. [acessado 2018 Out 10] 10.1016/j.socscimed.2017.03.029. 10.1016/j.socscimed.2017.03.029 28359581

[44] Assouad L, Chancel L, Morgan M (2018). Extreme inequality: evidence from Brazil, India, the Middle East, and South Africa. AEA Pap Proc.

[45] Greene W. Distinguishing between heterogeneity and inefficiency: stochastic frontier analysis of the World Health Organization's panel data on National Health Care Systems. New York University. 2003; 10.1002/hec.938. 10.1002/hec.938 15455464

[46] Herrera H, Pang G (2005). Efficiency of public spending in developing countries: an efficiency frontier approach. Policy research working paper no. 3645.

[47] Berger M, Messer J (2002). Public financing of health expenditure, insurance, and health outcomes. Appl Econ.

[48] Or Z (2000). Exploring the effects of health care on mortality across OECD countries, OECD labour market and social policy, Occasional Paper No 46.

[49] Verhoeven M, Gunnarsson V, Carcillo S (2007). Education and Health in G7 Countries: Achieving Better Outcomes with Less Spending, IMF Working Paper, No. 07/263.

[50] Mendes AN, Ianni AMZ, Marques MCC, Ferreira MJ, Silva THS (2017). A contribuição do pensamento da saúde coletiva à economia política da saúde. Saúde Soc.

[51] Elola J, Daponte A, Navarro V (1995). Health indicators and the organisation of health Care Systems in Western Europe. Am J Public Health.

[52] Franken M, Hoolman X (2013). Health system goals: a discrete choice experiment to obtain societal valuations. Health Policy.

[53] Rotarou ES, Sakellariou D. Neoliberal reforms in health systems and the construction of long-lasting inequalities in health care: a case study from Chile. Health Policy. 2017;121(5) [cerca de 9 p.]. [acessado 2018 Out 10] 10.1016/j.healthpol.2017.03.005. 10.1016/j.healthpol.2017.03.005 28385448

[54] Dhaoui I (2019). Healthcare system efficiency and its determinants: A two-stage Data Envelopment Analysis (DEA) from MENA countries. Working paper n 130. Economic Research Forum.

[55] Joumard I, André C, Nicq C. Health care systems: efficiency and institutions. Paris: Organisation for Economic Cooperation and Development, (OECD Economics Department Working Paper, 769); 2010. 10.1787/18151973.

[56] Lionel DT (2015). Determinants of health spending efficiency: a Tobit panel data approach based on DEA efficiency scores. Acta Univ Danubius.

[57] Ravangard R, Hatam N, Teimourizad A, Jafari A (2014). Factors affecting the technical efficiency of health systems: A case study of economic cooperation organization (ECO) countries (2004–10). Int J Health Policy Manag.

[58] Baudrillard J (1995). A Sociedade de Consumo, vol. 70.

[59] Pritchett D. Filmer L. Child mortality and public spending on health: how much does money matter. WPS 1864 (Policy Research Working Paper). World Bank Development Research Group Dec 1997 http://documents.worldbank.org/curated/en/885941468741341071/Child-mortality-and-public-spending-on-health-how-much-does-money-matter

[60] Avendano M, Kawachi I (2014). Why do Americans have shorter life expectancy and worse health than do people in other high-income countries?. Annu Rev Public Health.

[61] Harvey D. O novo imperialismo. São Paulo, Ed. Loyola, 2004.

[62] Lipovetsky G (2007). A felicidade paradoxal – Ensaio sobre a sociedade do hiperconsumo.

[63] Lencioni S, Acumulação primitiva: um processo atuante na sociedade contemporânea, Confins [Online], 14 2012, consultado o 08 novembro 2019. doi: 10.4000/confins.7424.

