# Supplementary material for: From income inequality to social inequity: impact on health levels in an international efficiency comparison panel
Source: BMC Public Health. 2021 Apr 8;21:688. doi: 10.1186/s12889-021-10395-7 (PMC8033748; doi:10.1186/s12889-021-10395-7)
Supplement: Supplementary file 1 — Additional file 1. Potential years gained in life expectancy at birth, sociocultural regions: comparison between methods (FE x DEA). This file depicts the amount of potential years gained in life expectancy at birth, calculated with both techniques used, permitting comparisons between countries, within sociocultural regions. Africa has been subdivided further, offering more detailed results. [file 12889_2021_10395_MOESM1_ESM.pdf]

Additional file 1– Potential years gained in life expectancy at birth, sociocultural regions: comparison between methods (FE x DEA)

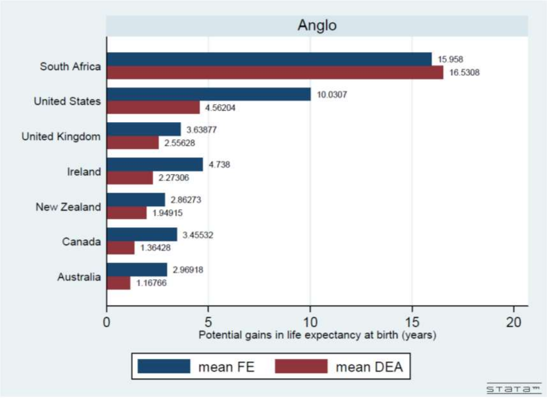

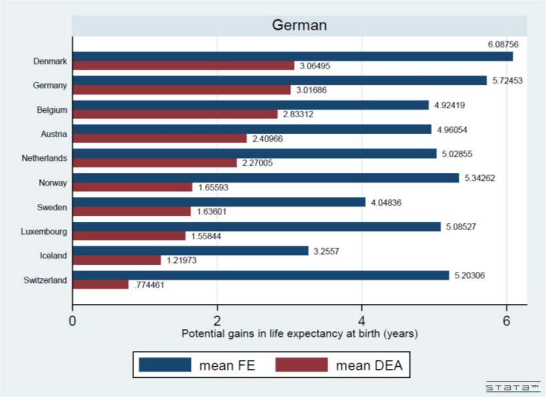

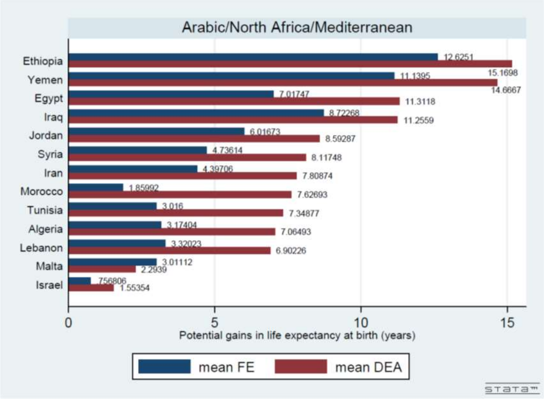

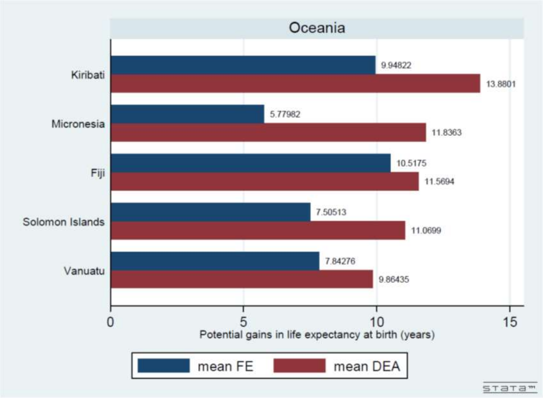

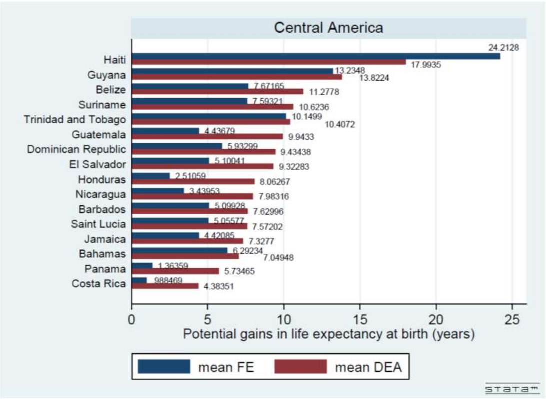

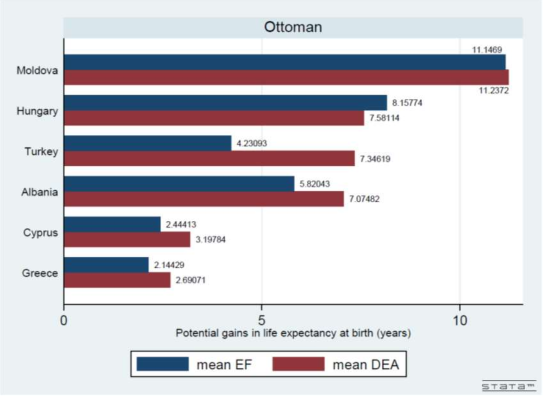

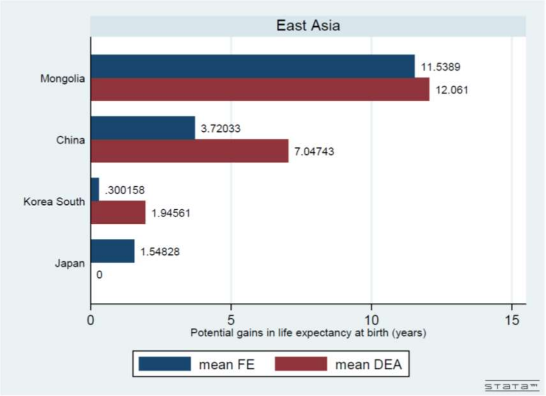

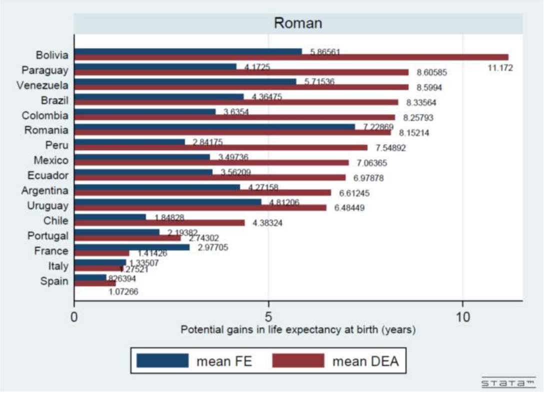

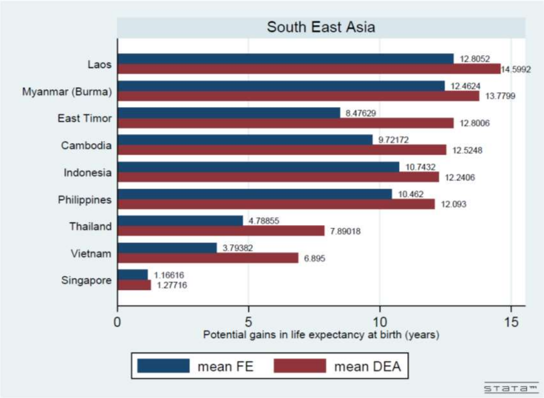
South East Asia
Laos
Myanmar (Burma)
East Timor
Cambodia
Indonesia
Philippines
Thailand
Vietnam
Singapore
12.8052
14.5992
12.4624
13.7799
8.47629
12.8006
9.72172
12.5248
10.7432
12.2406
10.462
12.093
4.78855
7.89018
3.79382
6.895
1.16616
1.27716
0
5
10
15
Potential gains in life expectancy at birth (years)
mean FE
mean DEA

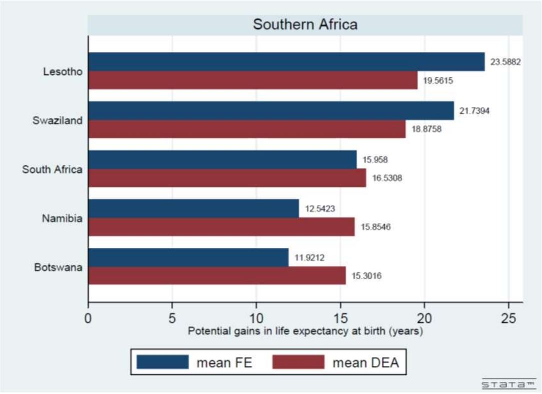
Southern Africa
Lesotho
Swaziland
South Africa
Namibia
Botswana
23.5882
19.5615
21.7394
18.8758
15.958
16.5308
12.5423
15.8546
11.9212
15.3016
0
5
10
15
20
25
Potential gains in life expectancy at birth (years)
mean FE
mean DEA

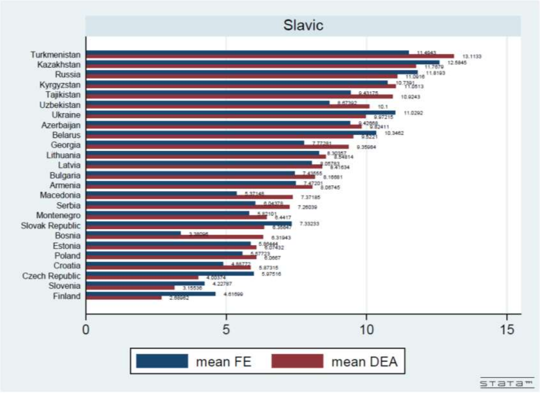
Slavic
Turkmenistan
Kazakhstan
Russia
Kyrgyzstan
Tajikistan
Uzbekistan
Ukraine
Azerbaijan
Belarus
Georgia
Lithuania
Latvia
Bulgaria
Armenia
Macedonia
Serbia
Montenegro
Slovak Republic
Bosnia
Estonia
Poland
Croatia
Czech Republic
Slovenia
Finland
0
5
10
15
mean FE
mean DEA

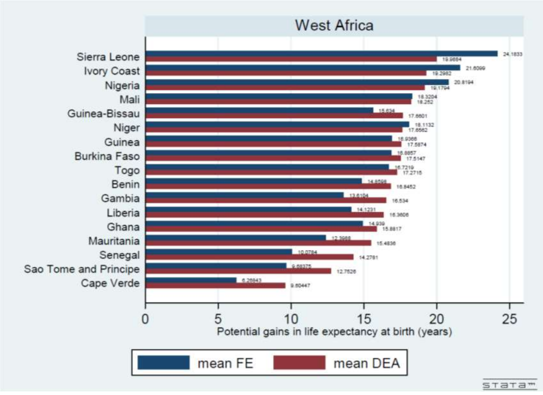
West Africa
Sierra Leone
Ivory Coast
Nigeria
Mali
Guinea-Bissau
Niger
Guinea
Burkina Faso
Togo
Benin
Gambia
Liberia
Ghana
Mauritania
Senegal
Sao Tome and Principe
Cape Verde
0
5
10
15
20
25
Potential gains in life expectancy at birth (years)
mean FE
mean DEA

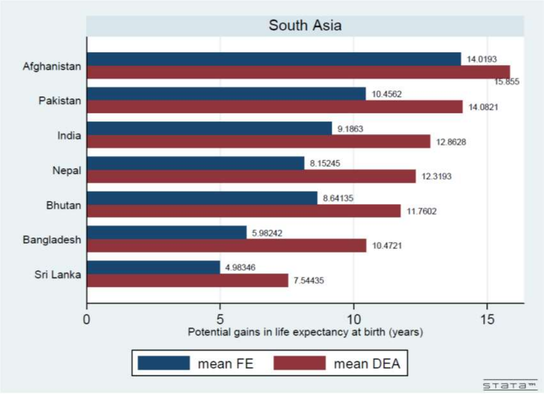
South Asia
Afghanistan
Pakistan
India
Nepal
Bhutan
Bangladesh
Sri Lanka
14.0193
15.855
10.4562
14.0821
9.1863
12.8628
8.15245
12.3193
8.64135
11.7602
5.98242
10.4721
4.98346
7.54435
0
5
10
15
Potential gains in life expectancy at birth (years)
mean FE
mean DEA

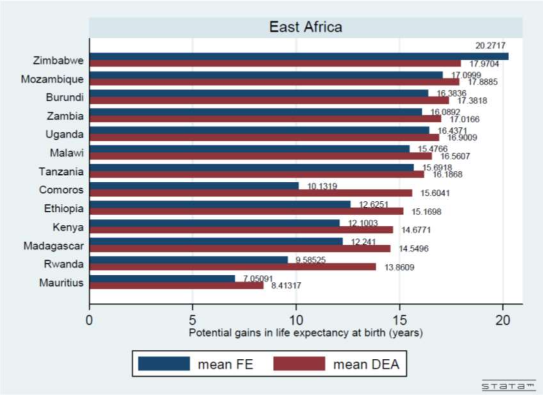
East Africa
Zimbabwe
Mozambique
Burundi
Zambia
Uganda
Malawi
Tanzania
Comoros
Ethiopia
Kenya
Madagascar
Rwanda
Mauritius
20.2717
17.9704
17.0999
17.8885
16.3836
17.3818
16.0892
17.0166
16.4371
16.9009
15.4766
16.5607
15.6918
16.1868
10.1319
15.6041
12.6251
15.1698
12.1003
14.6771
12.241
14.5496
9.58525
13.8609
7.05091
8.41317
0
5
10
15
20
Potential gains in life expectancy at birth (years)
mean FE
mean DEA

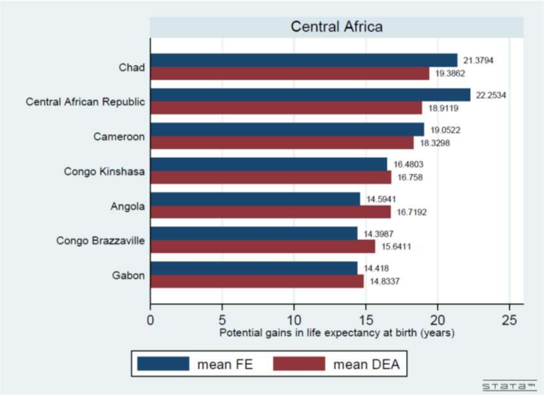
Central Africa
Chad
Central African Republic
Cameroon
Congo Kinshasa
Angola
Congo Brazzaville
Gabon
21.3794
19.3862
22.2534
18.9119
19.0522
18.3298
16.4803
16.758
14.5941
16.7192
14.3987
15.6411
14.418
14.8337
0
5
10
15
20
25
Potential gains in life expectancy at birth (years)
mean FE
mean DEA

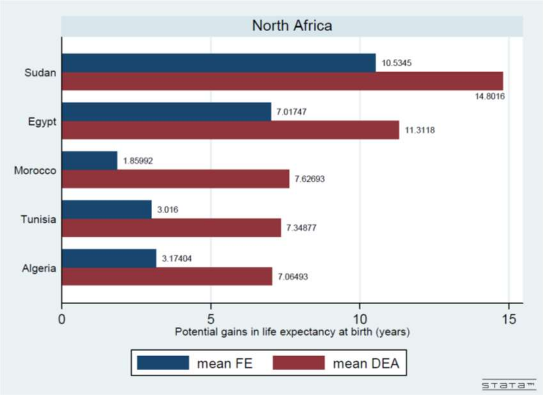
North Africa
Sudan
Egypt
Morocco
Tunisia
Algeria
10.5345
14.8016
7.01747
11.3118
1.85992
7.62693
3.016
7.34877
3.17404
7.06493
0
5
10
15
Potential gains in life expectancy at birth (years)
mean FE
mean DEA
