# Supplementary material for: From income inequality to social inequity: impact on health levels in an international efficiency comparison panel
Source: BMC Public Health. 2021 Apr 8;21:688. doi: 10.1186/s12889-021-10395-7 (PMC8033748; doi:10.1186/s12889-021-10395-7)
Supplement: Supplementary file 2 — Additional file 2. Potential reduction in infant mortality rates, sociocultural regions: comparison between methods (FE x DEA). This file presents the potential reduction in infant mortality rates, calculated with both techniques used, permitting comparisons between countries, within sociocultural regions. Africa has been subdivided further, offering more detailed results. [file 12889_2021_10395_MOESM2_ESM.pdf]

Additional file 2 – Potential reduction in infant mortality rates, sociocultural regions: comparison between methods (FE x DEA)

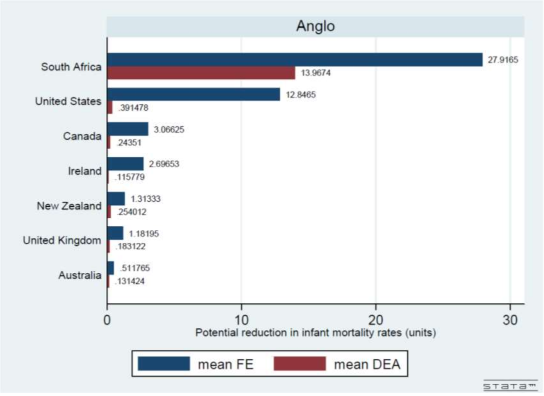

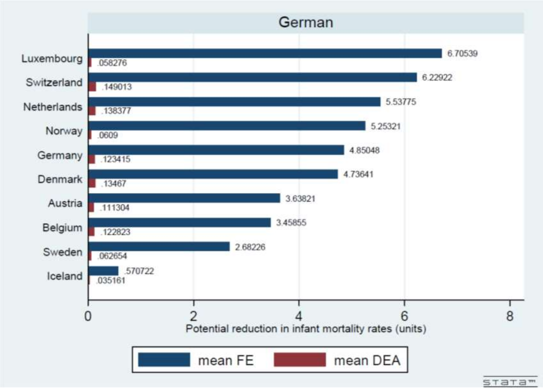

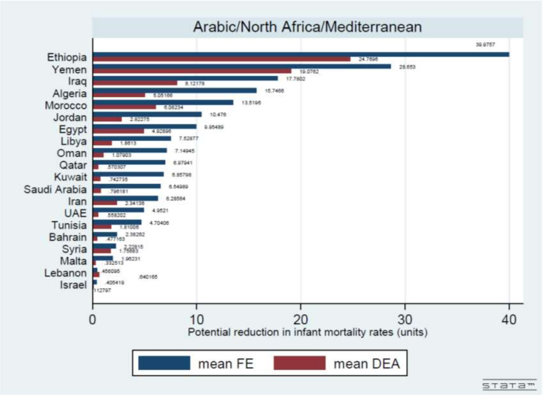

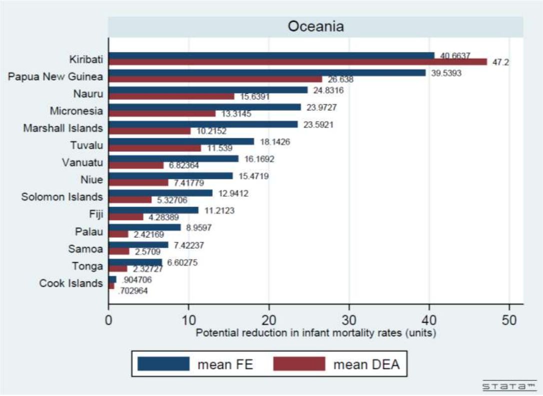

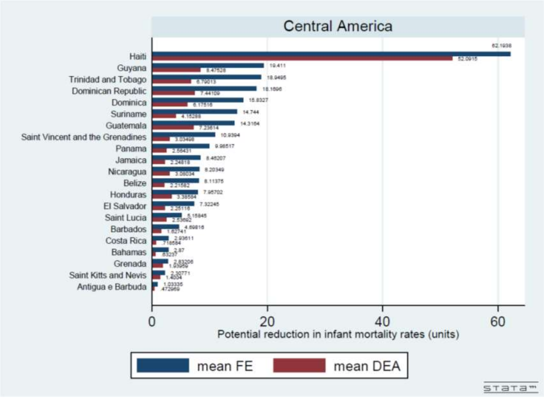

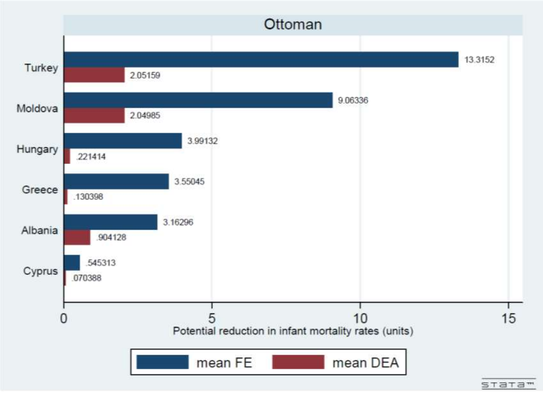

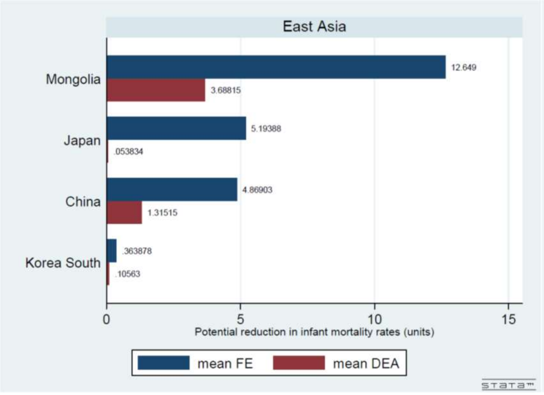

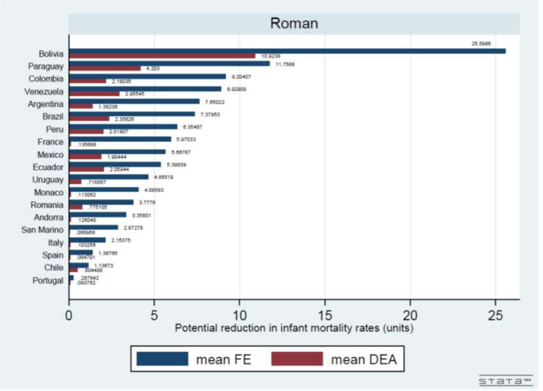

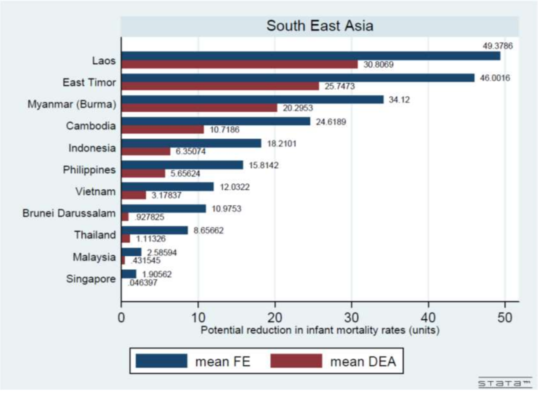
South East Asia
Laos
East Timor
Myanmar (Burma)
Cambodia
Indonesia
Philippines
Vietnam
Brunei Darussalam
Thailand
Malaysia
Singapore
49.3786
30.8069
46.0016
25.7473
34.12
20.2953
24.6189
10.7186
18.2101
6.35074
15.8142
5.65624
12.0322
3.17837
10.9753
.927825
8.65662
1.11326
2.58594
.431545
1.90562
.046397
0
10
20
30
40
50
Potential reduction in infant mortality rates (units)
mean FE
mean DEA

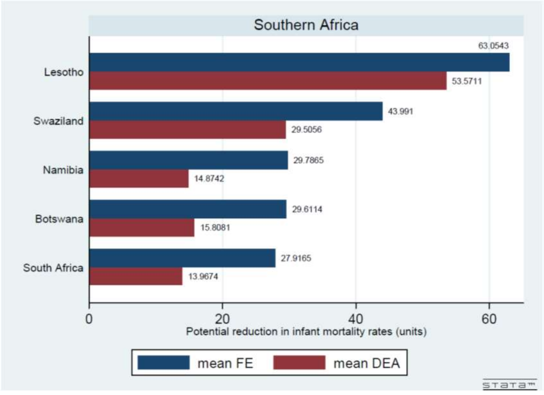
Southern Africa
Lesotho
Swaziland
Namibia
Botswana
South Africa
63.0543
53.5711
43.991
29.5056
29.7865
14.8742
29.6114
15.8081
27.9165
13.9674
0
20
40
60
Potential reduction in infant mortality rates (units)
mean FE
mean DEA

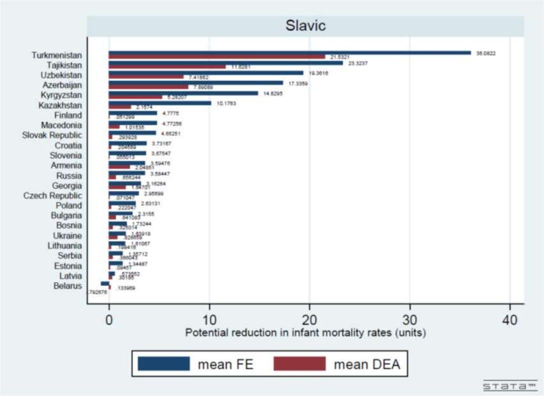
Slavic
Turkmenistan
Tajikistan
Uzbekistan
Azerbaijan
Kyrgyzstan
Kazakhstan
Finland
Macedonia
Slovak Republic
Croatia
Slovenia
Armenia
Russia
Georgia
Czech Republic
Poland
Bulgaria
Bosnia
Ukraine
Lithuania
Serbia
Estonia
Latvia
Belarus
0
10
20
30
40
Potential reduction in infant mortality rates (units)
mean FE
mean DEA

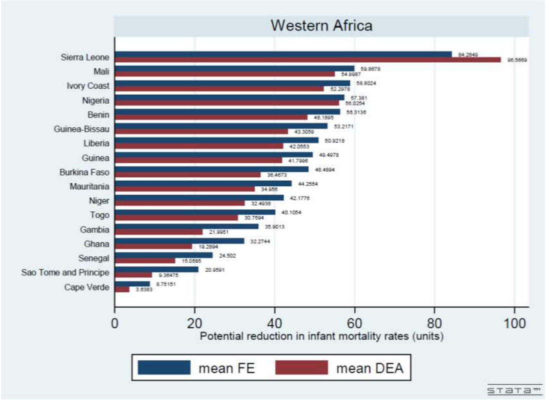
Western Africa
Sierra Leone
Mali
Ivory Coast
Nigeria
Benin
Guinea-Bissau
Liberia
Guinea
Burkina Faso
Mauritania
Niger
Togo
Gambia
Ghana
Senegal
Sao Tome and Principe
Cape Verde
0
20
40
60
80
100
Potential reduction in infant mortality rates (units)
mean FE
mean DEA

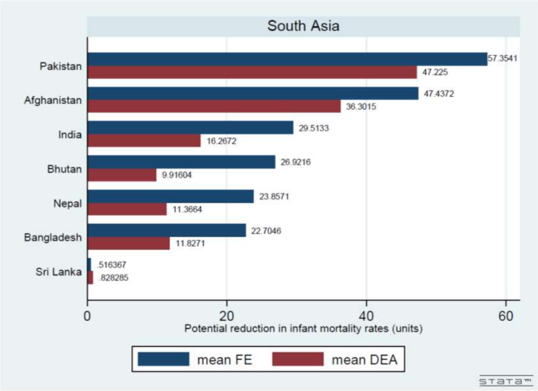
South Asia
Pakistan
Afghanistan
India
Bhutan
Nepal
Bangladesh
Sri Lanka
57.3541
47.225
47.4372
36.3015
29.5133
16.2672
26.9216
9.91604
23.8571
11.3664
22.7046
11.8271
.516367
.828285
0
20
40
60
Potential reduction in infant mortality rates (units)
mean FE
mean DEA

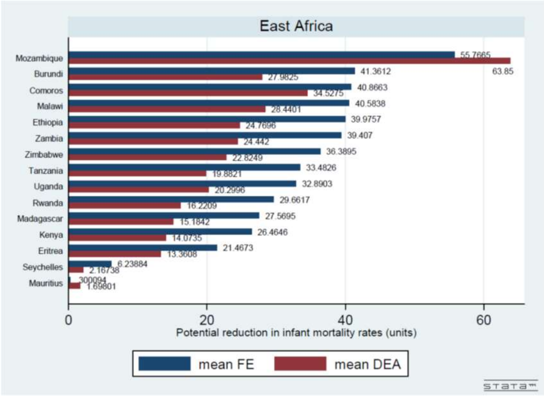
East Africa
Mozambique
Burundi
Comoros
Malawi
Ethiopia
Zambia
Zimbabwe
Tanzania
Uganda
Rwanda
Madagascar
Kenya
Eritrea
Seychelles
Mauritius
55.7665
63.85
41.3612
27.9825
40.8663
34.5275
40.5838
28.4401
39.9757
24.7696
39.407
24.442
36.3895
22.8249
33.4826
19.8821
32.8903
20.2996
29.6617
16.2209
27.5695
15.1842
26.4646
14.0735
21.4673
13.3608
6.23884
2.18738
.300094
1.69801
0
20
40
60
Potential reduction in infant mortality rates (units)
mean FE
mean DEA

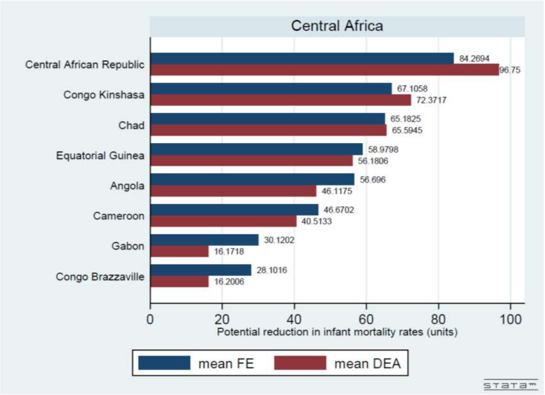
Central Africa
Central African Republic
Congo Kinshasa
Chad
Equatorial Guinea
Angola
Cameroon
Gabon
Congo Brazzaville
84.2694
96.75
67.1058
72.3717
65.1825
65.5945
58.9798
56.1806
56.696
46.1175
46.6702
40.5133
30.1202
16.1718
28.1016
16.2006
0
20
40
60
80
100
Potential reduction in infant mortality rates (units)
mean FE
mean DEA

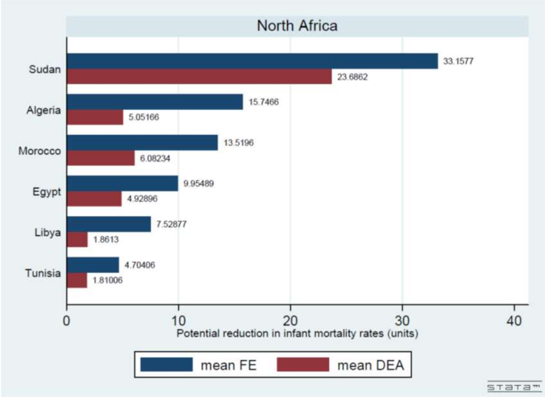
North Africa
Sudan
Algeria
Morocco
Egypt
Libya
Tunisia
33.1577
23.6862
15.7466
5.05166
13.5196
6.08234
9.95489
4.92896
7.52877
1.8613
4.70406
1.81006
0
10
20
30
40
Potential reduction in infant mortality rates (units)
mean FE
mean DEA
