# Supplementary material for: From income inequality to social inequity: impact on health levels in an international efficiency comparison panel
Source: BMC Public Health. 2021 Apr 8;21:688. doi: 10.1186/s12889-021-10395-7 (PMC8033748; doi:10.1186/s12889-021-10395-7)
Supplement: Supplementary file 3 — Additional file 3. DEA output-oriented efficiency scores, sociocultural regions: life expectancy at birth and infant mortality. This file exhibits figures of DEA efficiency scores, permitting comparisons between both outcome variables for countries, within sociocultural regions. Africa has been subdivided further, offering more detailed results. [file 12889_2021_10395_MOESM3_ESM.pdf]

Additional file 3 – DEA output-oriented efficiency scores, sociocultural regions: life expectancy at birth and infant mortality

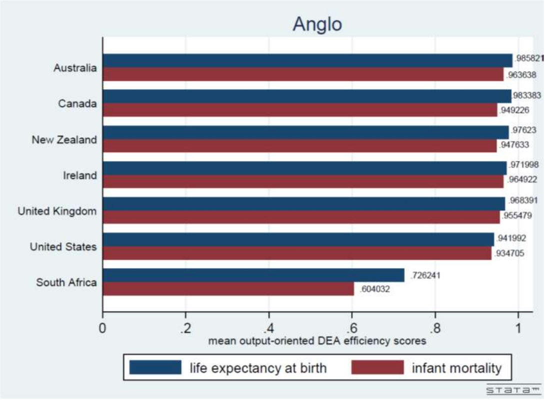

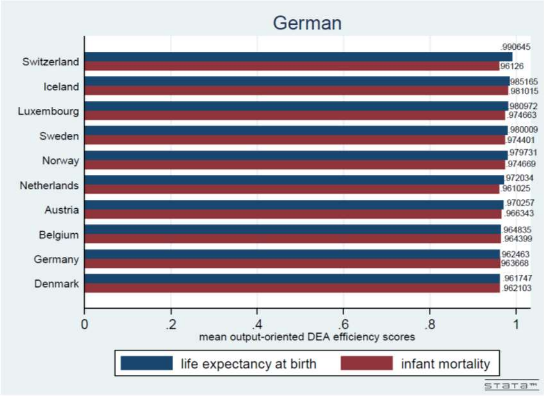

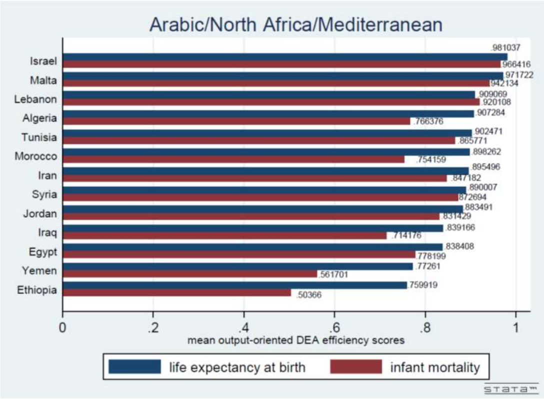

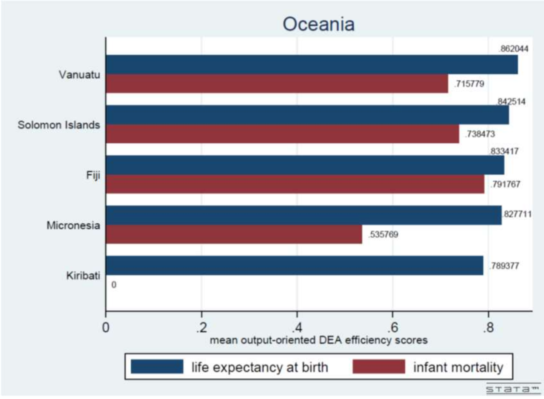

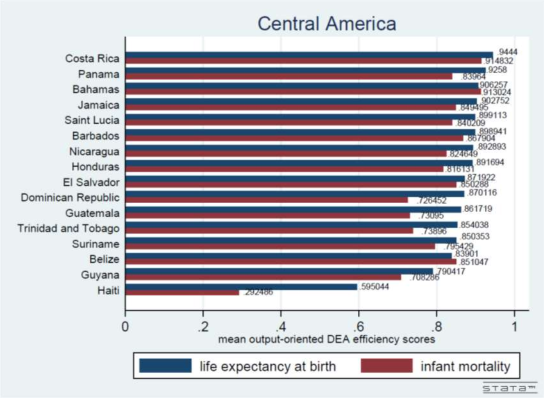

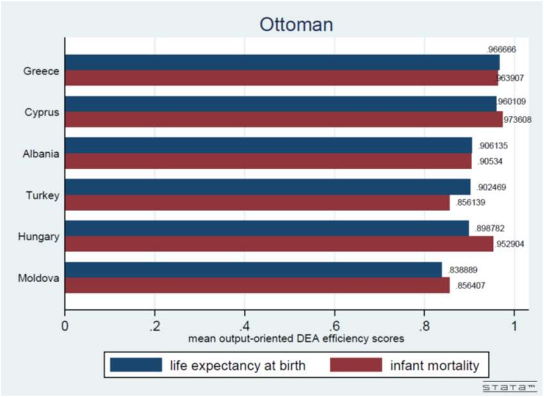

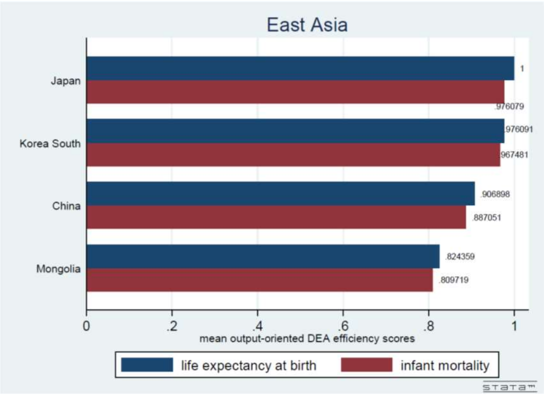

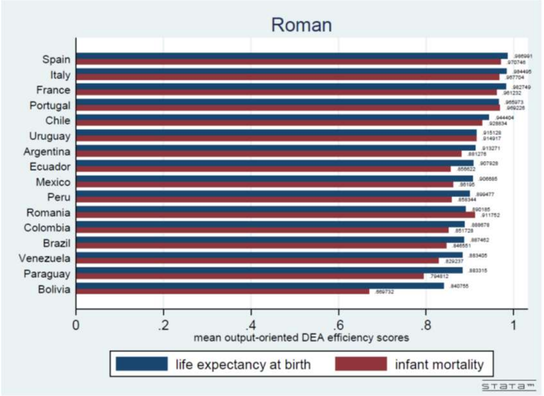

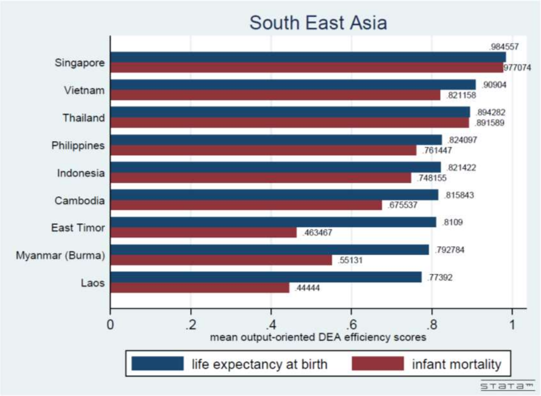
South East Asia
Singapore
Vietnam
Thailand
Philippines
Indonesia
Cambodia
East Timor
Myanmar (Burma)
Laos
.984557
.977074
.90904
.821158
.894282
.891589
.824097
.761447
.821422
.748155
.815843
.675537
.8109
.463467
.792784
.55131
.77392
.44444
0
.2
.4
.6
.8
1
mean output-oriented DEA efficiency scores
life expectancy at birth
infant mortality

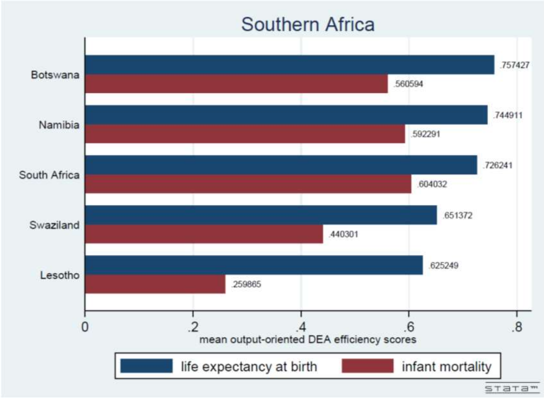
Southern Africa
Botswana
Namibia
South Africa
Swaziland
Lesotho
.757427
.560594
.744911
.592291
.726241
.604032
.651372
.440301
.625249
.259865
0
.2
.4
.6
.8
mean output-oriented DEA efficiency scores
life expectancy at birth
infant mortality

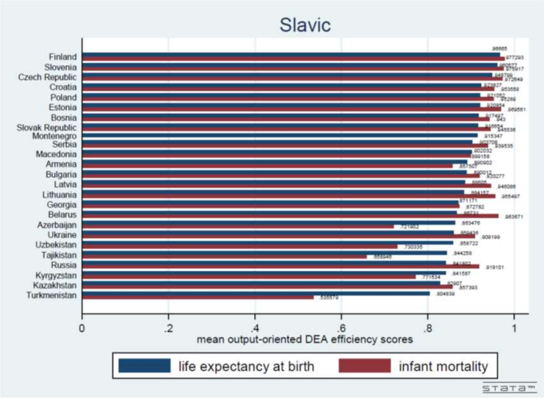
Slavic
Finland
Slovenia
Czech Republic
Croatia
Poland
Estonia
Bosnia
Slovak Republic
Montenegro
Serbia
Macedonia
Armenia
Bulgaria
Latvia
Lithuania
Georgia
Belarus
Azerbaijan
Ukraine
Uzbekistan
Tajikistan
Russia
Kyrgyzstan
Kazakhstan
Turkmenistan
0
.2
.4
.6
.8
1
mean output-oriented DEA efficiency scores
life expectancy at birth
infant mortality

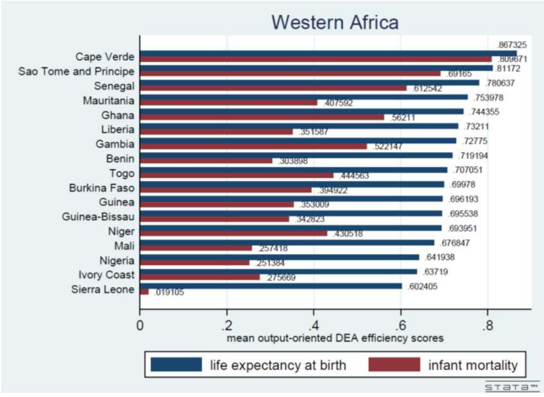
Western Africa
Cape Verde
Sao Tome and Principe
Senegal
Mauritania
Ghana
Liberia
Gambia
Benin
Togo
Burkina Faso
Guinea
Guinea-Bissau
Niger
Mali
Nigeria
Ivory Coast
Sierra Leone
.867325
.809671
.81172
.69165
.780637
.612542
.753978
.407592
.744355
.56211
.73211
.351587
.72775
.522147
.719194
.303898
.707051
.444563
.69978
.394922
.696193
.353009
.695538
.342823
.693951
.430518
.676847
.257418
.641938
.251384
.63719
.275669
.602405
.019105
0
.2
.4
.6
.8
mean output-oriented DEA efficiency scores
life expectancy at birth
infant mortality

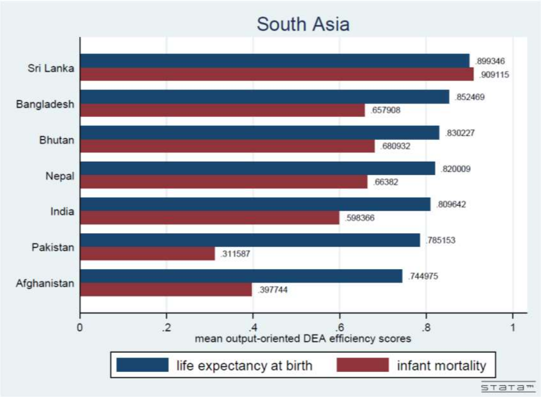
South Asia
Sri Lanka
Bangladesh
Bhutan
Nepal
India
Pakistan
Afghanistan
.899346
.909115
.852469
.657908
.830227
.680932
.820009
.66382
.809642
.598366
.785153
.311587
.744975
.397744
0
.2
.4
.6
.8
1
mean output-oriented DEA efficiency scores
life expectancy at birth
infant mortality

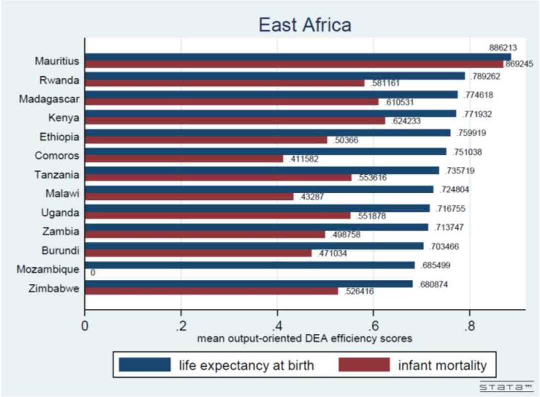
East Africa
Mauritius
Rwanda
Madagascar
Kenya
Ethiopia
Comoros
Tanzania
Malawi
Uganda
Zambia
Burundi
Mozambique
Zimbabwe
.886213
.869245
.789262
.581161
.774618
.610531
.771932
.624233
.759919
.50366
.751038
.411582
.735719
.553616
.724804
.43287
.716755
.551878
.713747
.498758
.703466
.471034
.685499
0
.680874
.526416
0
.2
.4
.6
.8
mean output-oriented DEA efficiency scores
life expectancy at birth
infant mortality

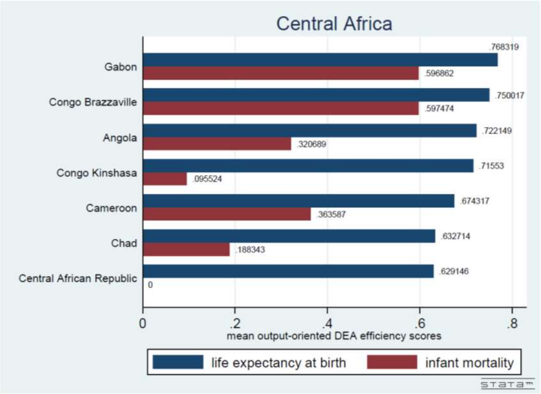
Central Africa
Gabon
Congo Brazzaville
Angola
Congo Kinshasa
Cameroon
Chad
Central African Republic
.768319
.596862
.750017
.597474
.722149
.320689
.71553
.095524
.674317
.363587
.632714
.188343
.629146
0
0
.2
.4
.6
.8
mean output-oriented DEA efficiency scores
life expectancy at birth
infant mortality

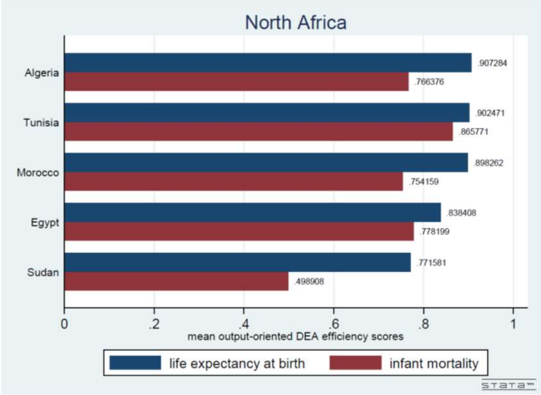
North Africa
Algeria
Tunisia
Morocco
Egypt
Sudan
.907284
.766376
.902471
.865771
.898262
.754159
.838408
.778199
.771581
.498908
0
.2
.4
.6
.8
1
mean output-oriented DEA efficiency scores
life expectancy at birth
infant mortality
